# Effect of AKT silence on malignant biological behavior of renal cell carcinoma cells

**DOI:** 10.1186/s12894-022-01087-4

**Published:** 2022-08-22

**Authors:** Zuan Li, DeYong Nong, Bincai Li, Haojian Wang, Chunlin Li, Zhi Chen, Ximing Li, Guihai Huang, Junhao Lin, Nan Hao, Wei Li

**Affiliations:** Department of Urology, The People’s Hospital of Guangxi Zhuang Autonomous Region, Nanning, China

**Keywords:** Renal cell carcinoma, AKT, Silence, Biological behavior

## Abstract

**Background:**

As the most common malignant tumor of primary renal tumor, renal cell carcinoma (RCC) is the highly invasive disease with high mortality. AKT is a serine/threonine kinase that play a critical role in the phosphoinositide 3-kinase (PI3K) signaling pathway, and it is an attractive target for RCC treatment. The aim of present study was to investigate the effect of AKT silence on malignant behavior of renal cell carcinoma cells.

**Methods:**

AKT expression was quantified by immunohistochemistry in tumor tissues and normal tissues. The human RCC cell lines Caki-2 cell were chosen for this study. The optimal silencing siRNA was subsequently selected by RT-qPCR and western blot. The effect of AKT silence on RCC cells was investigated by CCK8 assay, transwell assay, scratch test and flow cytometry. The AKT1 expression in human renal cell carcinoma tissue was detected by immunohistochemical staining.

**Results:**

The AKT in Caki-2 cells was silenced successfully. The results shown AKT silence could inhibit cell proliferation, invasion, and, migration. In addition, AKT silence could promote Caki-2 cell apoptosis with prevention of RCC cells move from G1 phase to S phase. Immunohistochemical staining revealed significant difference of expression of AKT1 in RCC tissues and normal renal tissues. Taken together, AKT family members might involve in malignant growth of RCC, and might be a potential therapeutic target.

**Conclusion:**

Our data show that AKT silence inhibited cell proliferation, invasion, and, migration of Caki-2 cell, and promoted Caki-2 cell apoptosis. Moreover, AKT silence prevented RCC cells move from G1 phase to S phase. Therefore, AKT may act as an effective therapeutic target for RCC.

**Supplementary Information:**

The online version contains supplementary material available at 10.1186/s12894-022-01087-4.

## Background

Worldwide, renal cell carcinoma (RCC) is one of the most common malignant tumor in the urinary system and approximately account 90% for kidney cancer. The global morbidity and mortality of RCC increased by 2–3% every decade [1]. About 70% of patients were diagnosed with early-stage RCC which can be cured by surgery, but 30% of patients present with metastatic RCC at the time of diagnosis [2]. Furthermore, according to the statistics of the American survival study in RCC, the 5-year overall survival rate of patients with early-stage RCC was 82.4%. However, the 5-year survival rate was 5–20% in advanced patients, which lacking effective treatment [3]. Currently, surgery, targeted therapies and immunological therapy were common options for advanced disease [4], however, drug resistance and adverse events limited its treatment efficiency, many patients suffered recurrence eventually. Therefore, it is significance to investigate the molecular mechanisms of RCC, which will help to identify potential therapeutic targets and new prognostic markers for RCC.

AKT, 57-kDa serine/threonine kinase, also known as protein kinase B (PKB), is a critical mediator of growth factor-induced cell survival [5]. AKT can modulate the function of many downstream proteins involved in cellular survival, proliferation, migration, metabolism, and angiogenesis [6]. Structurally, AKT is composed of three domains: An amino-terminal (N-terminal), a central and a carboxyl-terminal fragment (C-terminal) [7]. To date, three isoforms have been isolated, named AKT1, AKT2 and AKT3, and the different isoforms are believed to mediate critical functions in cancer pathophysiology. For example, AKT1 promotes mammary tumorigenesis in mouse models, AKT2 promotes breast cancer cell migration and invasion in vitro [8]. Moreover, it has been demonstrated that specific AKT isoforms drive particular cancers. For example, AKT2, but not AKT1, mediates survival and maintenance of PTEN-deficient prostate cancer [9]. So AKT family members were considered as attractive targets for cancer therapy.

The most common RCC type is clear cell RCC (ccRCC) which account for 70–80% of RCC. It is well known that loss of Von Hippel Lindau (VHL) gene and upregulation of hypoxia-inducible factors (HIF) promote multiple growth factors in ccRCC [10]. The VHL/HIF interacted with PI3K/AKT pathway through a complex signaling network which contributing to RCC tumorigenesis [11]. However, exact function of AKT in RCC still remain unclear. In present study, AKT of RCC cells was knocked down, and a set of cellular function assays were performed, considering the role of AKT1 on tumorigenesis, The expression of AKT1 in renal cell carcinoma tissue was detected by immunohistochemical staining. The findings might help to clarify the roles of AKT family members in RCC.

## Methods

### Immunohistochemistry

Total 38 cases samples (17 cases of RCC and 21 cases of normal kidney tissue) were collected from The People’s Hospital of Guangxi Zhuang Autonomous Region. Informed consent was obtained from all patients. The specimens embedded in paraffin were cut into sections, then slides were dewaxed with dimethylbenzene and rehydrated through grade alcohols. After the addition of citrate buffer for antigen retrieval under pressure, the slides were placed in 3% H_2_O_2_ for 15 min. Then the sample were incubated with primary antibodies against AKT1 (ab238477, Abcam, 1/1000) at 4 °C overnight. After washed with PBS, the slides were cultured in the secondary antibody at room temperature for 30 min. Then washed in PBS, slides were incubated with DAB for 5 min and counterstained with hematoxylin, dehydrated and mounted. The study was approved by the local Ethics Committee (The People’s Hospital of Guangxi Zhuang Autonomous Region). The research complies with the provisions of the Declaration of Helsinki (as revised in 2013).

### Cell culture

The caki-2 cell lines were purchased form the Chinese Academy of Science (Shanghai, China). The cells were propagated in McCoy’s 5a medium supplemented with 10% fetal bovine serum (E510002, Sangon Biotech) containing penicillin–streptomycin solution (100×) (P1400, Solarbio, China) at incubator with 37 °C with 5% CO_2_. Finally, cells exhibiting good growth at the logarithmic growth phase were selected for transfection.

### Cell grouping and transfection

According to the AKT sequence information in NCBI, 3 interference sequences were constructed (Table 1) by Sangon Biotech (shanghai) Co., Ltd. Caki-2 cells were collected and seeded into a 6-well culture plates and then assigned into 5 groups respectively: control group, negative control (NC) group (transfected with empty plasmid negative control), siAKT-1 group (transfected with AKT-siRNA-1), siAKT-2 group (transfected with AKT-siRNA-2), siAKT-3 group (transfected with AKT-siRNA-3). Prior to cell transfection, the cells exhibiting good growth condition were seeded in a 6-well plate at the density of 5 × 10^5^ cells. When cell coverage rate reached 70%, the cells were transfected with the medium replaced with a serum-and -antibiotic-free medium. 125 ul Opti-MEN (31985-062, Gibco) was added to two EP tube, 5 ul Lipofectamine 3000 (L3000015, Invitrogen) was added to one tube and 0.25 nmol siRNA was added to the other tube, and two tubes were incubated at room temperature for 5 min after mixed. Then the two EP tubes were mixed and incubated at room temperature for 15 min. The mixed liquid was dropped into the corresponding well in the 6-well plate, and cells was put back into the incubator for culture. After 4–6 h of transfection, 1 ml of complete medium containing 20% serum was added into the 6-well plate. At 48 h after transfection, the cells were collected for subsequent experiments.

**Table 1:** siRNA sequence

siRNA	Sequence
AKT-siRNA-1	GCUAUUGUGAAGGAGGGUUTT AACCCUCCUUCACAAUAGCTT
AKT-siRNA-2	GCACCUUCAUUGGCUACAATT UUGUAGCCAAUGAAGGUGCTT
AKT-siRNA-3	GGAGACUGACACCAGGUAUTT AUACCUGGUGUCAGUCUCCTT
NC	UUCUCCGAACGUGUCACGUTT ACGUGACACGUUCGGAGAATT

### RT-qPCR

Total RNA was extracted using ultrapure RNA Extraction Kit (CW0581M, CWBIO). Total RNA was reversely transcribed to cDNA using HiScript II Q RT SuperMix for qPCR (+ gDNA wiper) (R223-01, Vazyme). PCR was performed by using 2 × SYBR Green PCR Master Mix (A4004M, Lifeint). The reaction conditions were as follows: pre-denaturation at 95 °C for 10 min and 40 cycles of denaturation at 95 °C for 10 s, annealing at 58 °C for 30 s, and extending at 72 °C for 30 s. Data were analyzed using the ana2−ΔΔCT method. The primer sequences for AKT were as follows: AKT sense, forward 5′GCTATTGTGAAGGAGGGTTGG3′ and reverse 5′ACAGTCTGGATGGCGGTTG3′. β-actin sense, forward 5′TGGCACCCAGCACAATGAA′ and reverse 5′CTAAGTCATAGTCCGCCTAGAAGCA3′.

### Western blot

Caki-2 cells transfected with siRNA were lysed with protein lysate RIPA (C1053, Applygen Technologies INC.). After centrifugation for 10 min at 12,000 r/min, the supernatant was collected, and the total protein concentration was determined with the BCA protein assay kit (CW0014S, CWBIO) and preserved at − 20 °C. Then total protein was loaded to 10% sodium dodecyl sulfate (151-21-3, XiLong Scientific)-polyacrylamide gel electrophoresis (A1010, Solarbio) (SDS-PAGE). Finally transfer to a polyvinylidene fluoride (PVDF) (IPVH00010, Millipore) membrane at constant pressure of 60 V was performed. Membrane blockade was conducted with 5% skim milk powder and then incubated with a primary antibody for overnight reaction at 4 °C. After washing, the membrane was incubated for 2 h at room temperature with horseradish peroxidase (HRP)-conjugated secondary antibodies. The super sensitive enhanced chemiluminescence (ECL) enlight-plus (RJ239676, Thermo Fisher scientific) reaction solution was applied for developing purposes. Then images were acquired using a Chemi DocTM XRS+ imaging system (Bio-Rad). The information of antibodies was listed as follow: Internal control primary antibody: Mouse Monoclonal Anti-GAPDH (TA-08, ZSGB-BIO, 1/2000), secondary antibody: HRP-labeled goat anti-mouse IgG (H + L) (ZB-2305, ZSGB-BIO, 1/2000), purpose primary antibody: Rabbit Anti AKT (ab8805, Abcam, 1/500), secondary antibody: HRP-labeled goat anti-rabbit IgG (H + L) (ZB-2301, ZSGB-BIO, 1/2000).

### CCK8 assay

After transfection, cells were seeded into 96-well plates at a density of 7 × 10^3^cells/well and cultured for 24 h. Then 10 μl of CCK8 solution (KGA317, KEYGEN) was added directly to each well and incubated for 2 h at 37 °C. The absorbance was measured at 450 nm.

### Transwell assay

Matrigel (BD, USA) was selected for transwell assay. The medium in the well was removed, then stained with 0.1% crystal violet (G1061, Solarbio) for 1 h after washed by PBS for 5 min. Cells in the inner compartment were scraped off by a moist cotton swab, then chamber inverted on slide was photographed. Then 1 ml of 33% acetic acid was added in every well to dissolved staining fluid in cells after crystal violet was removed, and 200 μl of cell suspension was added to 96-well plates. The optical density value (OD value) at 570 nm was measured using a multi-scan spectrum (S/N502000011, TECAN).

### Scratch test

Parallel lines at 0.5 cm intervals was marked at the back of 6-well plate, then cells were evenly inoculated into the 6-well plate. When cell coverage rate reached 90%, wounds were created using 200 μl pipette tip. After washed three times by PBS the cells were incubated with a serum-free medium at 37 °C in a 5% CO_2_ incubator. The cells were photographed at the 0 h and 48 h time points.

### Flow cytometry

After transfection for 48 h, the cells (1 × 10^6^–3 × 10^6^) were washed twice with PBS and centrifuged at 1500 rpm for 3 min, and re-suspended in cold 1× binding buffer (300 μl). Then 3 μl of Annexin-V-FITC and 5 μl of PI (AP101-100-kit, MULTI SCIENCES) were added to each tube. After staining for 10 min at room temperature, 200 μl of 1× binding buffer was added to each tube. The apoptosis was analyzed using a flow cytometer (NovoCyte 2060R, ACEA Biosciences Inc.).

After transfection for 48 h, the cell suspension was centrifuged at 1500 rpm for 3 min and supernatant was removed. The cells were then added in 1 ml of PBS and centrifuged at 1500 rpm for 3 min. Then 1 ml of DNA Staining solution and 10 μl of Permeabilization solution (CCS102, MULTI SCIENCES) were added to each tube, and the mixtures were oscillated for 10 s by vortex mixer (XH-C, Changzhou LANGYUE instruments Maunfacturing Co., Ltd.). Cell cycle was measured using a flow cytometer (NovoCyte 2060R, ACEA Biosciences Inc.).

### Statistical analyses

All the statistical analyses were conducted using GraphPad Prism 6.0 and IBM SPSS 19.0 software. Data were presented as mean ± standard deviation (SD), and *p* < 0.05 was considered statistically significant.

## Results

### AKT1 was overexpression in RCC

**Fig. 1:**
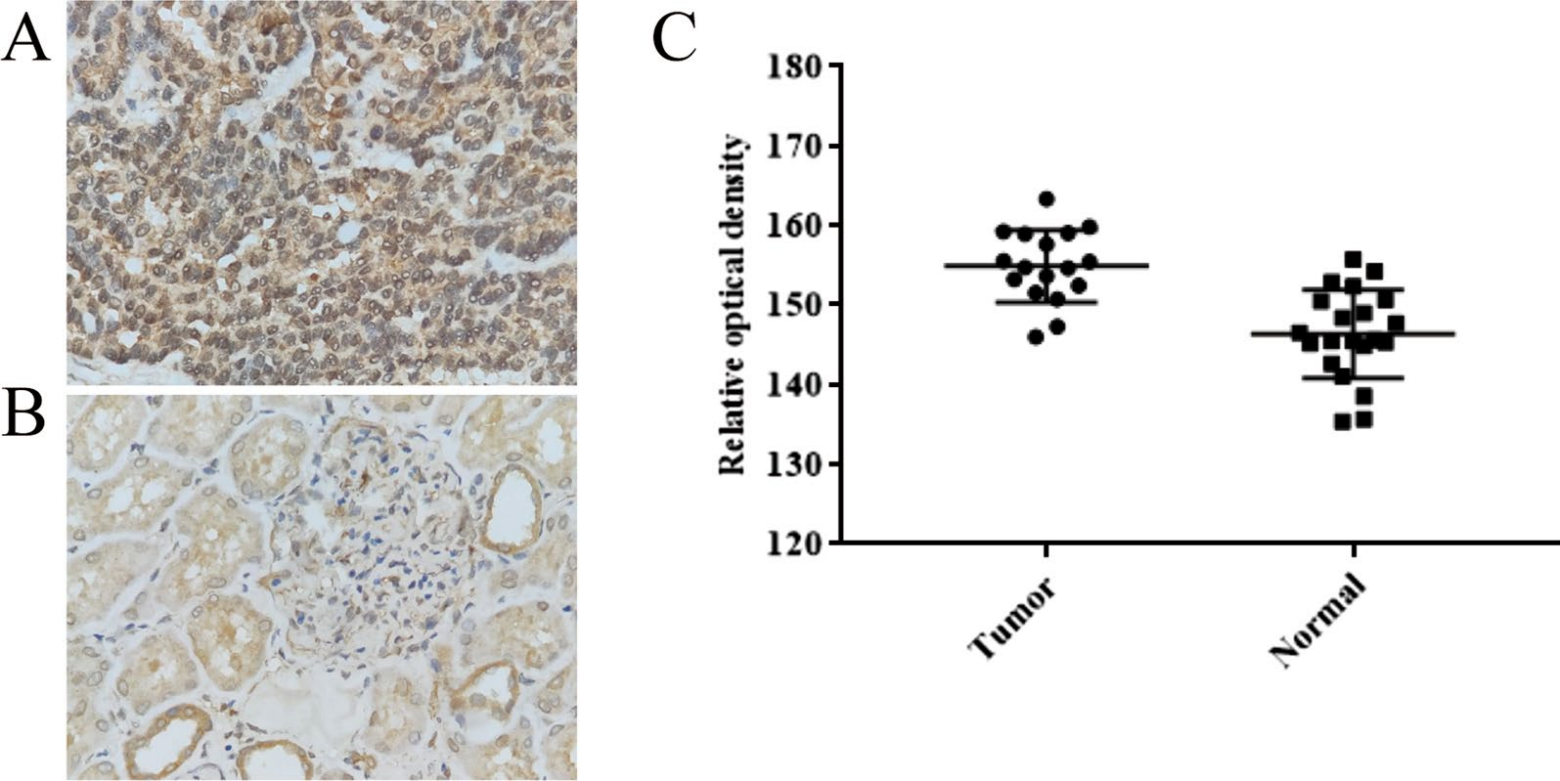
Higher positive expression rate of AKT1 was found in RCC tissues. **a** the positive expression of AKT1 in RCC tissues; **b** the low expression of AKT1 was found in adjacent normal tissues. Results of immunohistochemistry was shown with 400× magnification respectively. **c** The relative optical density of tumor and normal tissues (*p* < 0.05)

As one of isoform of AKT, AKT1 could suppress cancer cell invasion, migration and accelerate the onset of tumorigenesis which had described above, so we explored the expression of AKT1 in this section. The protein expression of AKT1 in tumor tissues and normal kidney tissues was detected by immunohistochemical staining. The significant difference staining intensity was observed (Fig. 1a–c and Table2). As shown in Fig. 1c, the relative optical density (ROD) of tumor tissues was significantly higher than normal tissues which had significantly difference according to statistical analysis (*p* < 0.001). In summary, these findings showed elevated expression of AKT1 in tumor tissues compared with normal tissues.

**Table 2:** Relative optical density (ROD) of AKT1 in tumor and normal tissues

	N (%)	Mean value of ROD	*p* value*
Tumor tissues	17 (44.7)	154.883	< 0.001
Normal tissues	21 (55.3)	146.326	

*Tumor tissues versus normal tissues

### RT-qPCR and western blot verify the effectiveness of interference vector

**Fig. 2:**
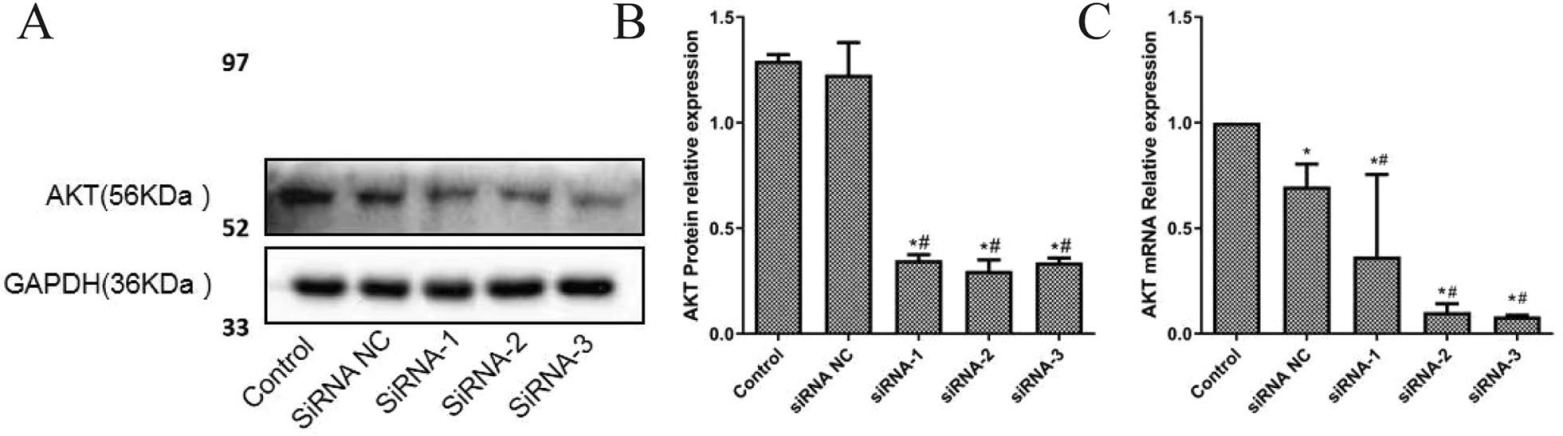
SiRNA-2 had the best jamming effect of the three vectors. **a** protein bands of 5 groups; **b** the protein expressions of AKT in 5 groups examined by western blot analysis; **c** the mRNA expression of AKT in 5 groups examined by RT-qPCR. Results were presented as mean ± SD. *p < 0.05, compared with control group, ^#^*p* < 0.05, compared with NC group

Three interference vectors were constructed then transfected into Caki-2 cells. RT-qPCR and western blot were used to determine an appropriate vector for subsequent experiments. The results (Fig. 2a–c) of RT-qPCR demonstrated that SiRNA-2 had the best jamming effect of the three vectors. And compared with the control group and the NC group, three siRNA group displayed significantly reduced expression of AKT. The results of western blot show that the protein expression of AKT for three siRNA group was significantly decreased compared with control group and the NC group. According to the above analysis, siRNA-2 group was identified for subsequent experiments.

### AKT silence inhibits RCC cell proliferation

**Fig. 3:**
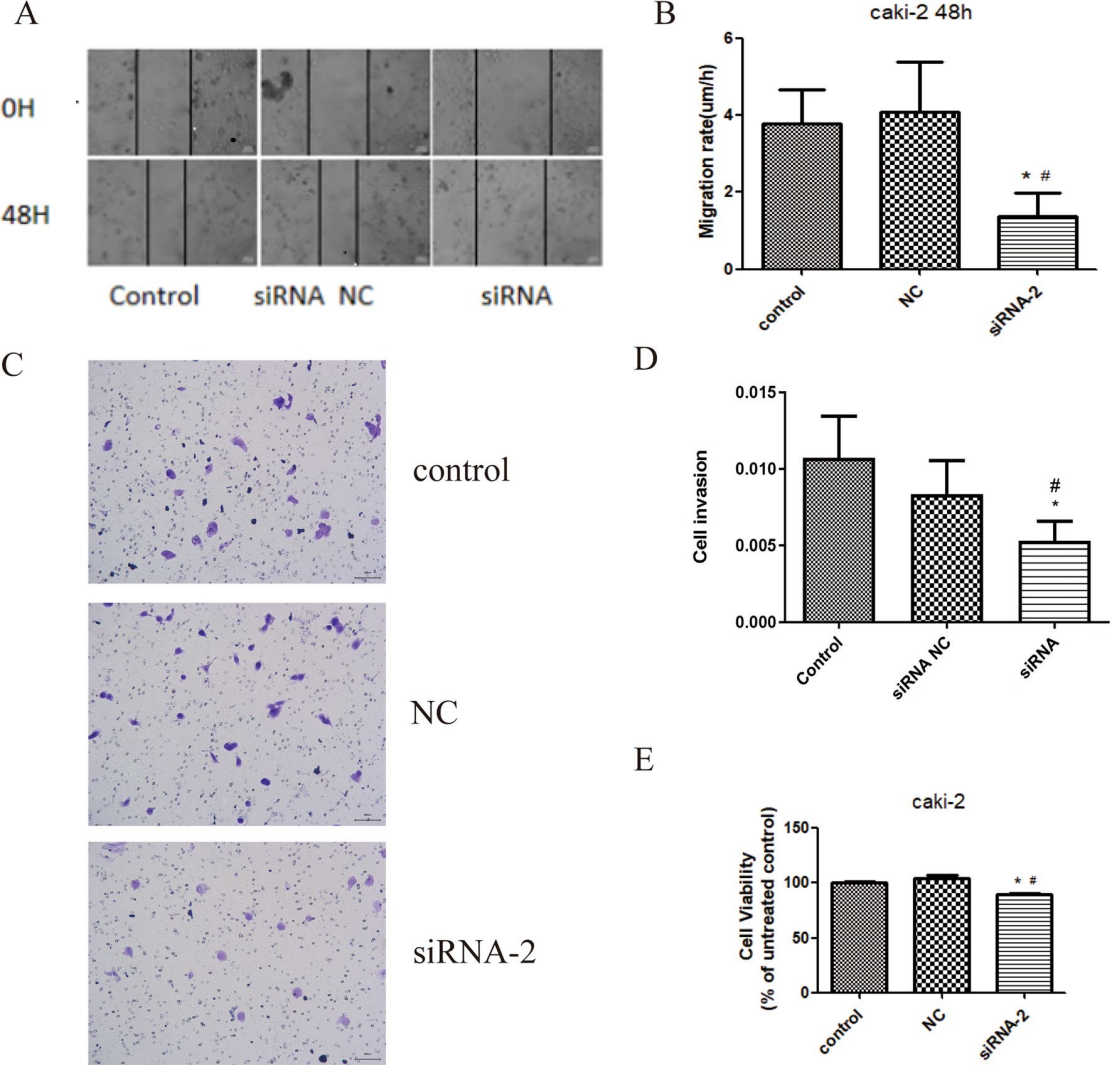
SiRNA-2 inhibited cell proliferation, migration, and invasion. **a** and **b** Scratch test to determine cell migration. **c** and **d** Transwell assay to determine cell invasion. **e** CCK8 method determine cell proliferation. Results were presented as mean ± SD. *p < 0.05, compared with control group, ^#^*p* < 0.05, compared with NC group

Inhibiting cell proliferation is important strategy for the treatment of tumors. To determine the impact of AKT silence on RCC cell proliferation, we used the CCK8 method for proliferation assay. As shown in Fig. 3e, the cell viability of Caki-2 cells transfected with siRNA was decreased (*p* < 0.05). These results suggested that AKT might promote the proliferation of Caki-2 cells.

### AKT silence inhibits Caki-2 cell migration

Cell migration is an important biological characteristic of tumor cells. Therefore, we investigated the effects of AKT silence on migration by scratch test. The results (Fig. 3a, b) revealed that the average migration rate at 48 h after scratch was: 3.78% in the control group, 4.10% in the NC group, and 1.36% in the siRNA group. Compared with both the control and NC groups, the migration rate of siRNA group was significantly reduced (*p* < 0.05). From the above results, it was possible that AKT silence could inhibit Caki-2 cell migration.

### AKT silence suppresses invasive ability of Caki-2 cells

Inhibiting invasion is the key procedure for cancer control. Transwell assay was employed to help detect cell invasion, and the results (Fig. 3c, d) showed that there was no significant difference in cell invasion between the control group and the NC group. Compared with the control group and the NC group, the siRNA group displayed greatly low cell density with decreased invasion ability (*p* < 0.05). These results suggested that AKT silence significantly reduced the invasive ability of the Caki-2 cells.

### AKT silence promotes cell apoptosis and Caki-2 cycle arrest

**Fig. 4:**
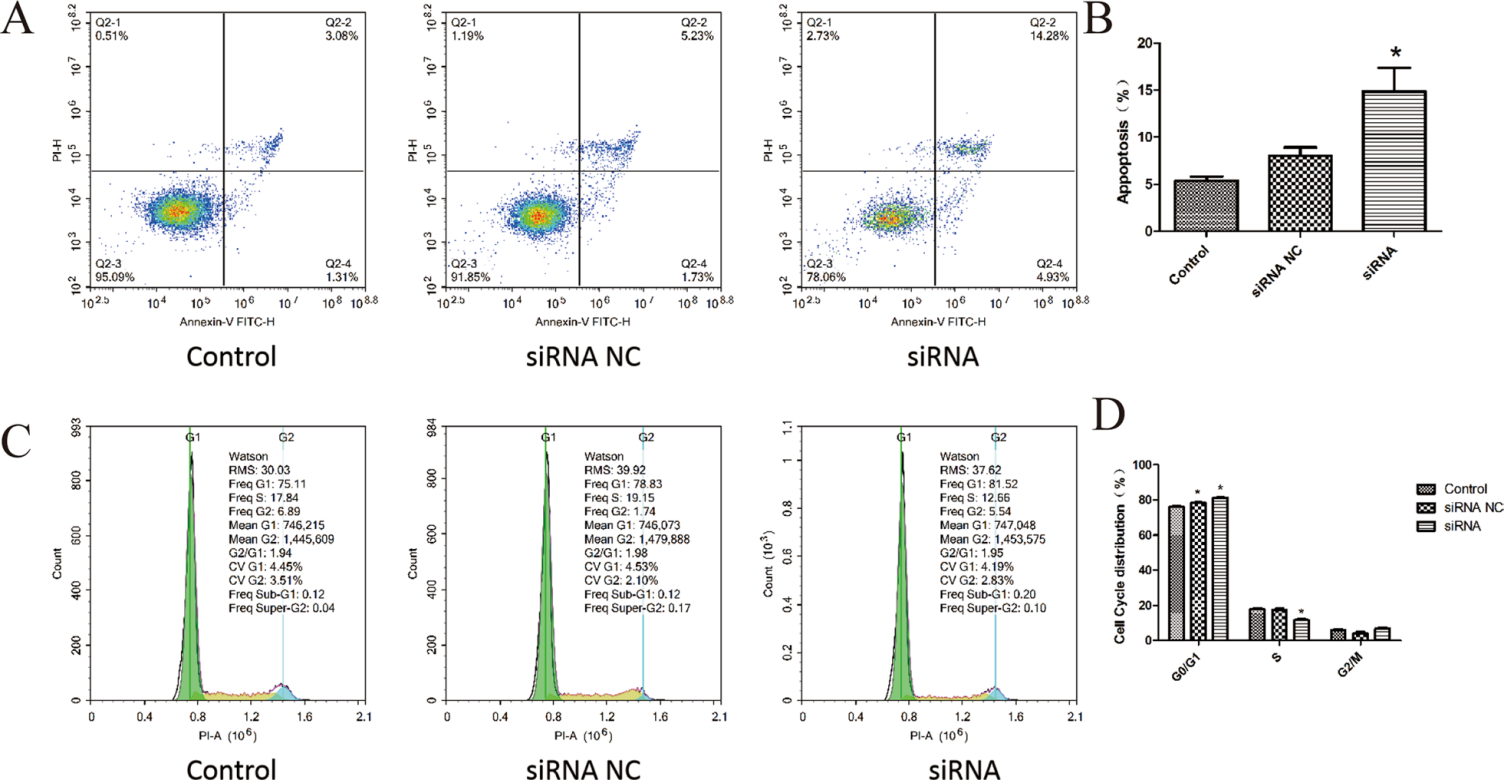
AKT silence enhanced cell apoptosis and caused cell cycle arrest. **a** and **b** Flow cytometry detection of cell apoptosis. **c** and **d** Flow cytometry detection of cell cycle. Results were presented as mean ± SD. *p < 0.05, compared with control group

Flow cytometry was applied to examine Caki-2 cell apoptosis. The apoptotic rates of the control group and the NC group were 5.34% and 8.04%, respectively (Fig. 4a, b). Compared with the control group and the NC group, the siRNA group exhibited an upward trend in cell apoptosis (*p* < 0.05), the apoptotic rates of Caki-2 cells transfected with the siRNA was 14.88%. Based on our observations we concluded that AKT silence enhanced the apoptosis Caki-2 cell.

Flow cytometry was also performed to evaluate the cell cycle, the results (Fig. 4c, d) of which revealed there to be no significant difference regarding cell cycle distribution between the control group and the NC group. Compared with the control group and the NC group, the siRNA group displayed suppressed cell proportion at S phase (*p* < 0.05), and promoted cell proportion at the G0/G1 phase. There was no significant difference among the cells at the G2 phase between all the groups (*p* > 0.05). The above findings suggested that AKT silence can arrest cells at the G0/G1 phase.

## Discussion

As an highly invasive disease, RCC exhibits intrinsic insensitivity to chemotherapy and radiation therapy, the surgery remaining the most effective treatment for early stage and local advanced RCC [12]. When RCC develop into metastatic stage, the mean survival rate of patients is limited. The treatment of metastatic RCC remain a challenge for the urologist. Despite the metastasis was correlated with morbidity and survival of RCC, the knowledge of molecular mechanisms of RCC metastasis was not known well yet [13]. AKT, an member in the PI3K pathway, playing a key role in human malignancies [14]. Previous research shown the overexpression of AKT is a common molecular characteristic of malignancies. Expression of certain oncogenes or loss of cancer suppressor genes can result in activation of the PI3K/AKT signaling pathway. For example, the mutations of EGFR/PI3K, or the loss of PTEN, as well as mutations of AKT itself can result in increased PI3K/AKT signaling [15]. Therefore, AKT is considered to be an attractive target for cancer therapy.

As a signaling molecules of cell growth and differentiation, the deregulation of AKT plays a key role in the pathogenesis of many cancers. Previous studies indicated that many type of cancer were associated with the overexpression of AKT. High expression levels of the phosphorylated AKT protein were determined in esophageal squamous cell carcinoma compared with normal esophageal mucosa [16]. Moreover, The association between overexpression or amplification of AKT and ovarian cancer has been revealed [17]. So it was widely accepted that p-AKT played an important role in malignant aggression, however, the correlation between p-AKT expression and prognosis of RCC remains controversial. Increased p-AKT levels was positive correlated with higher grade and stage of RCC [18], however, another study draw an quiet different conclusion, high nuclear p-AKT expression was associated with a favorable prognosis [19]. In present study, we evaluated the function of AKT and AKT1 in renal cell carcinoma. In addition, it was reported that the expression of AKT was positively correlated with the expression of p-AKT [20], which suggested AKT and p-AKT had the similar research value. In present study the antibody of total AKT was used in cellular experiments, based on the findings of in vitro experiments and previous studies, AKT1 was involved in regulating cell growth and proliferation, so we explored expression of AKT1 in human tissues by IHC staining. Similar to the previous research, we found that AKT1 exhibited up-regulated expression in RCC tissues compared with normal tissues. Studies had shown that AKT activation enhance chemical carcinogenesis in vivo. Wu et al. found that overexpression of AKT1 accelerates carcinogen-induced tumorigenesis in tumor mouse models [21]. Furthermore, the cooperation of overexpression of AKT-E17K and urethane induced in mouse lung epithelial cells [22]. These results show that overexpression of AKT is sufficient for carcinogenesis.

Generally speaking, cells migration and invasion are necessary for cancer metastasis. Xu et al. asserted that AKT is activated by phosphorylation on Thr308 or Ser473 and this phosphorylation could influence a variety of cell biological functions, such as cell cycle, cell growth, proliferation, apoptosis, and migration [23]. Broustas et al. reported that RAD9 modulates AKT activation and affected cell migration and invasion in prostate cancer [24]. RUNX2 reactivates PI3K/AKT pathways, providing the high metastatic potential to melanoma cells [25]. Despite above findings, the biological function of AKT in RCC still remains unclear. In order to identify the biological role of AKT, we used AKT silencing RCC cell lines to investigate its tumorigenic properties in RCC metastasis. CCK-8 assay demonstrated that AKT silence inhibited CCK-8 viability. Subsequently, the effect of AKT silence on cell motility was detected using scratch test and transwell assay. The results suggested that the number of invasive cells in siRNA group was significantly reduced lower, and the migrated distance of siRNA group was significantly decreased. In summary, AKT silence significantly inhibited Caki-2 cells proliferation, migration and invasion, which suggested that AKT might promote RCC metastasis.

Apoptosis is one of natural defenses against cancer, and the ability to escape from apoptosis is a feature of tumor [26]. Previous studies had demonstrated that AKT could block apoptosis by inhibit pro-apoptotic proteins, such as BAD, caspase-9 and fork head family [27]. Additionally, AKT could phosphorylate and activate numerous oncogenic proteins of cycle progression, such as MDM2 and E3 ligase [28, 29]. Therefore, we further examined the apoptosis and cell cycle analysis by flow cytometry on cells of AKT silence. We observed that AKT silence could arrest cells at the G0/G1 phase and promotes Caki-2 cell apoptosis, which might be potential mechanism of inhibition in proliferation assay. We found AKT silence could decreased malignant behavior of Caki-2 cells. Zhu et al. found that acylglycerol kinase promotes the progression of RCC via activating the PI3K/AKT/GSK3β signaling pathway [30]. A previous study demonstrated that MiR-182 inhibited RCC cell proliferation, apoptosis, and invasion by regulating PI3K/AKT/mTOR signaling pathway [31]. As so far, the molecular mechanism of AKT on the development and metastasis of RCC was still not entirely elucidated, and it should be the focus of further research.

## Conclusions

The experimental results of the current study suggested that AKT silence inhibited cell proliferation, invasion and migration in Caki-2 cells, as well as promoted Caki-2 cell apoptosis by preventing cells move from G1 phase to S phase. The results of immunohistochemical staining partially confirmed the findings of invitro experiments. In conclusion, AKT may act as an potential therapeutic target for RCC.

## Supplementary Information

**Additional file 1**. Data and analysis of this study.

## Data Availability

All data generated and analysis during this study are included in this published article and its Additional file 1.

## Abbreviations

RCC: Renal cell carcinoma
AKT: A serine/threonine kinase
PI3K: Phosphoinositide 3-kinase
RT-qPCR: Real-time quantitative polymerase chain reaction
ccRCC: Clear cell RCC
PBS: Phosphate buffered saline
DAB: Diaminobenzidine
NCBI: National Center for Biotechnology Information
RIPA: Radio immunoprecipitation assay
SDS-PAGE: Sodium dodecyl sulfate-polyacrylamide gel electrophoresis
BCA: Bicinchoninic acid
PVDF: Polyvinylidene fluoride
ECL: Enhanced chemiluminescence
CCK-8: Cell counting kit-8
OD: Optical density value
FITC: Fluorescein lsothiocyanate
PI: Propidium iodide
