# Supplementary material for: Effect of AKT silence on malignant biological behavior of renal cell carcinoma cells
Source: BMC Urol. 2022 Aug 22;22:129. doi: 10.1186/s12894-022-01087-4 (PMC9396790; doi:10.1186/s12894-022-01087-4)
Supplement: Supplementary file 1 — Additional file 1. Data and analysis of this study. [file 12894_2022_1087_MOESM1_ESM.zip › Supplementary/Legends for supplementary tables.docx]

**Legends for supplementary tables**

**Table S1-3 data analysis of CCK8.** SiRNA-2 inhibited cell proliferation.

**Table S4-6 data analysis of apoptosis.** AKT silence enhanced cell apoptosis.

**Table S7-9 data analysis of cell cycle.** AKT silence caused cell cycle arrest.

**Table S10-12 data analysis of PCR.** SiRNA-2 had the best jamming effect of the three vectors.

**Table S13-15 data analysis of Scratch test.** AKT silence enhanced cell migration.

**Table S16-20 data analysis of Transwell.** AKT silence enhanced cell invasion.

**Table S21-22 data analysis of Western blot.** SiRNA-2 had the best jamming effect of the three vectors.
