# Supplementary figures and images for: Effect of AKT silence on malignant biological behavior of renal cell carcinoma cells
Source: BMC Urol. 2022 Aug 22;22:129. doi: 10.1186/s12894-022-01087-4 (PMC9396790; doi:10.1186/s12894-022-01087-4)

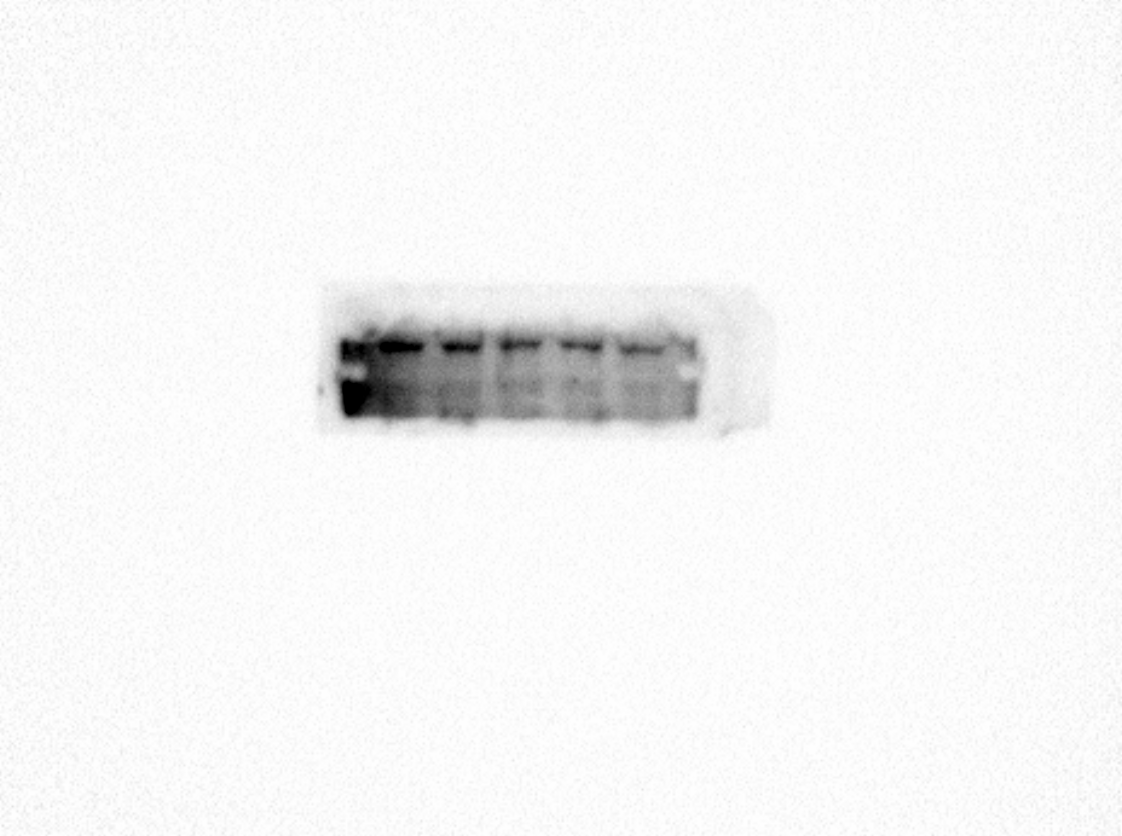

Supplement: Supplementary file 1 — Additional file 1. Data and analysis of this study. [file 12894_2022_1087_MOESM1_ESM.zip › Supplementary/AKT image 1.tif]

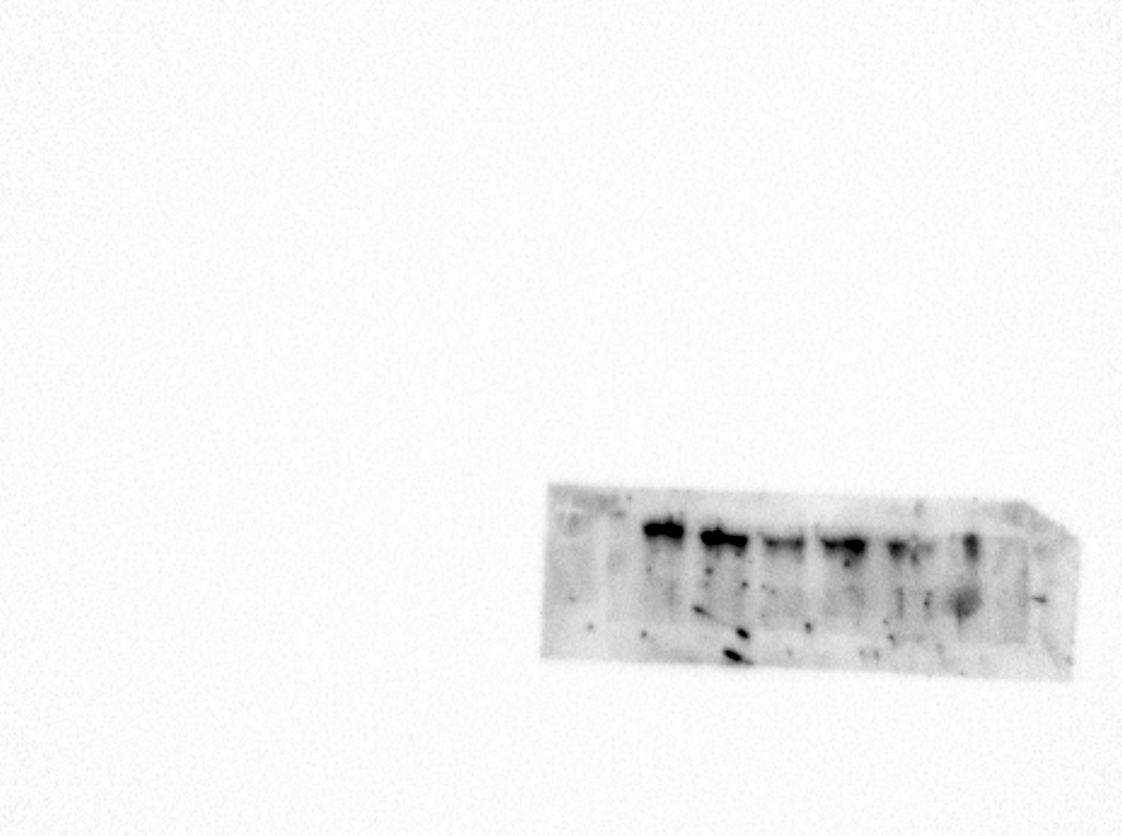

Supplement: Supplementary file 1 — Additional file 1. Data and analysis of this study. [file 12894_2022_1087_MOESM1_ESM.zip › Supplementary/AKT image 2.tif]

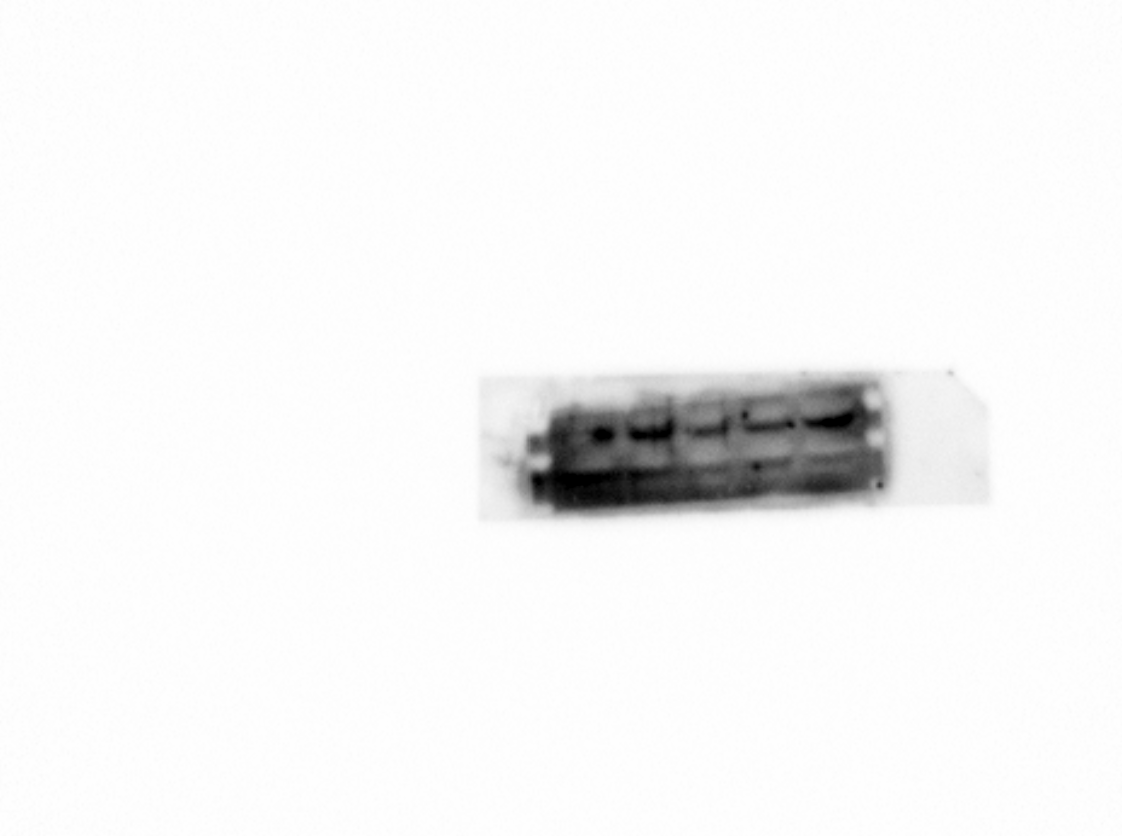

Supplement: Supplementary file 1 — Additional file 1. Data and analysis of this study. [file 12894_2022_1087_MOESM1_ESM.zip › Supplementary/AKT image 3.tif]

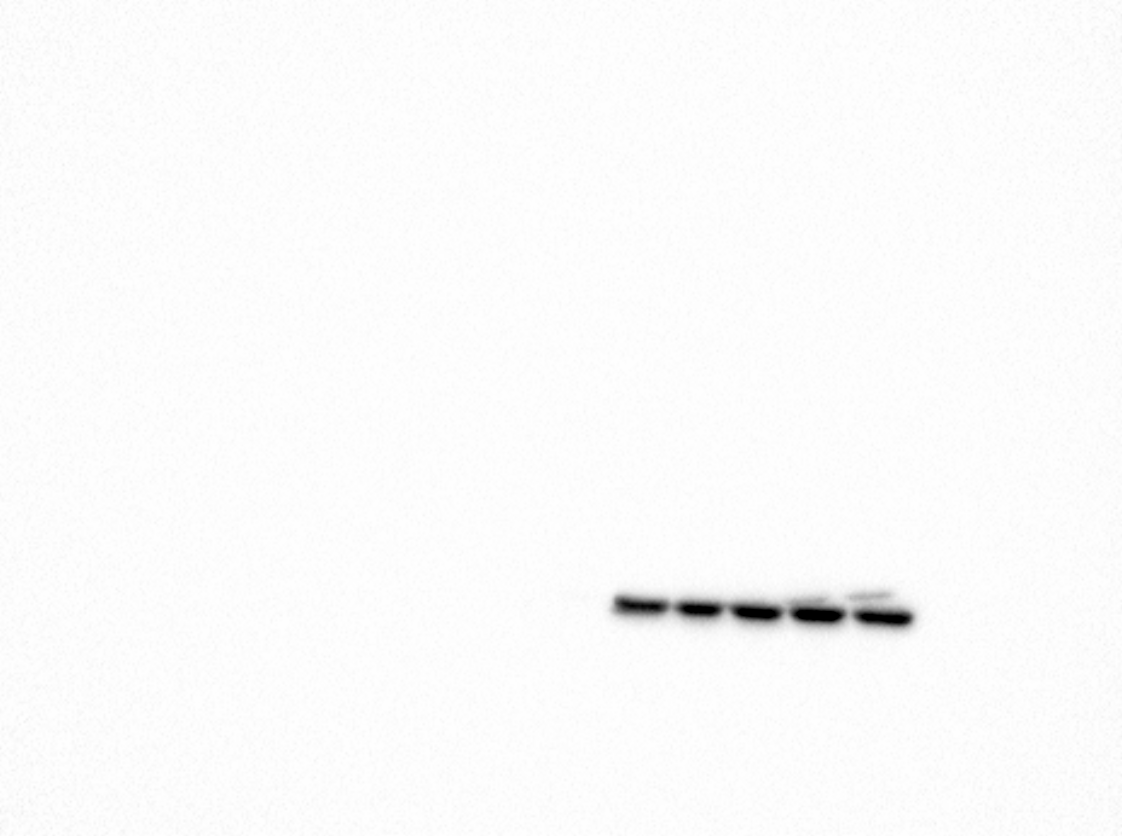

Supplement: Supplementary file 1 — Additional file 1. Data and analysis of this study. [file 12894_2022_1087_MOESM1_ESM.zip › Supplementary/GAPDH 1.tif]

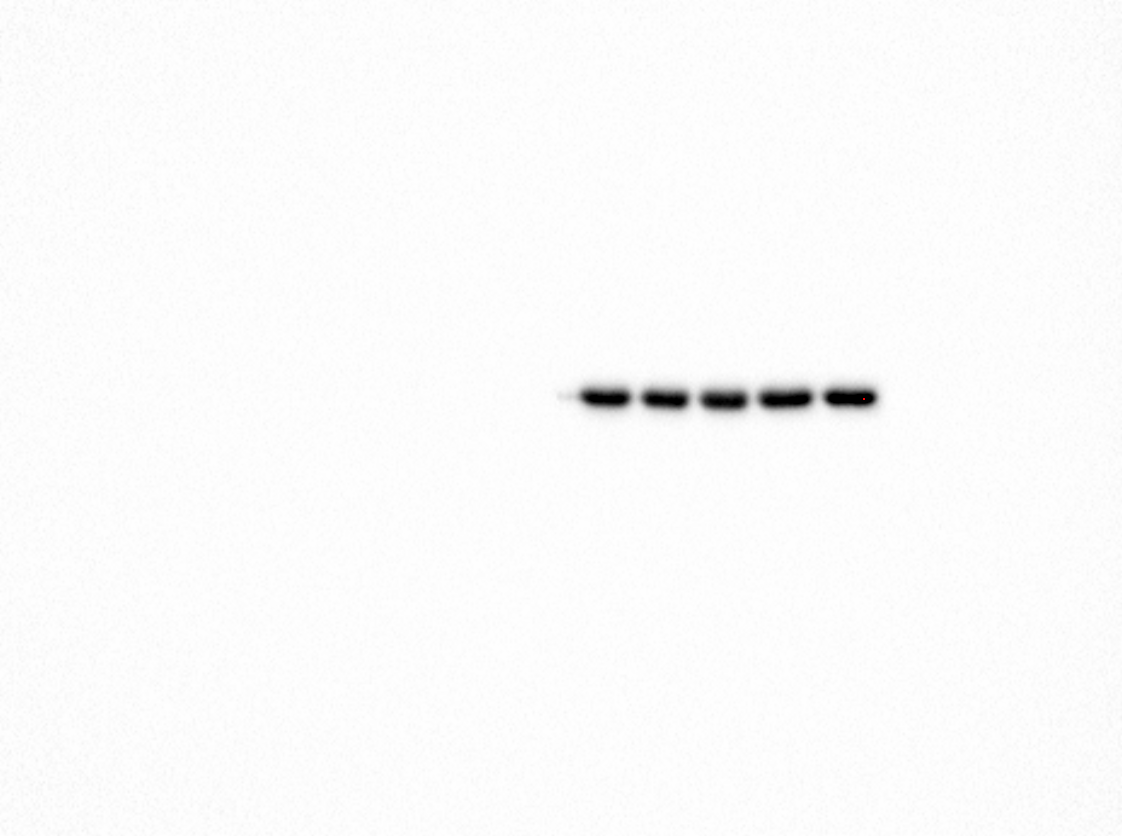

Supplement: Supplementary file 1 — Additional file 1. Data and analysis of this study. [file 12894_2022_1087_MOESM1_ESM.zip › Supplementary/GAPDH 2.tif]

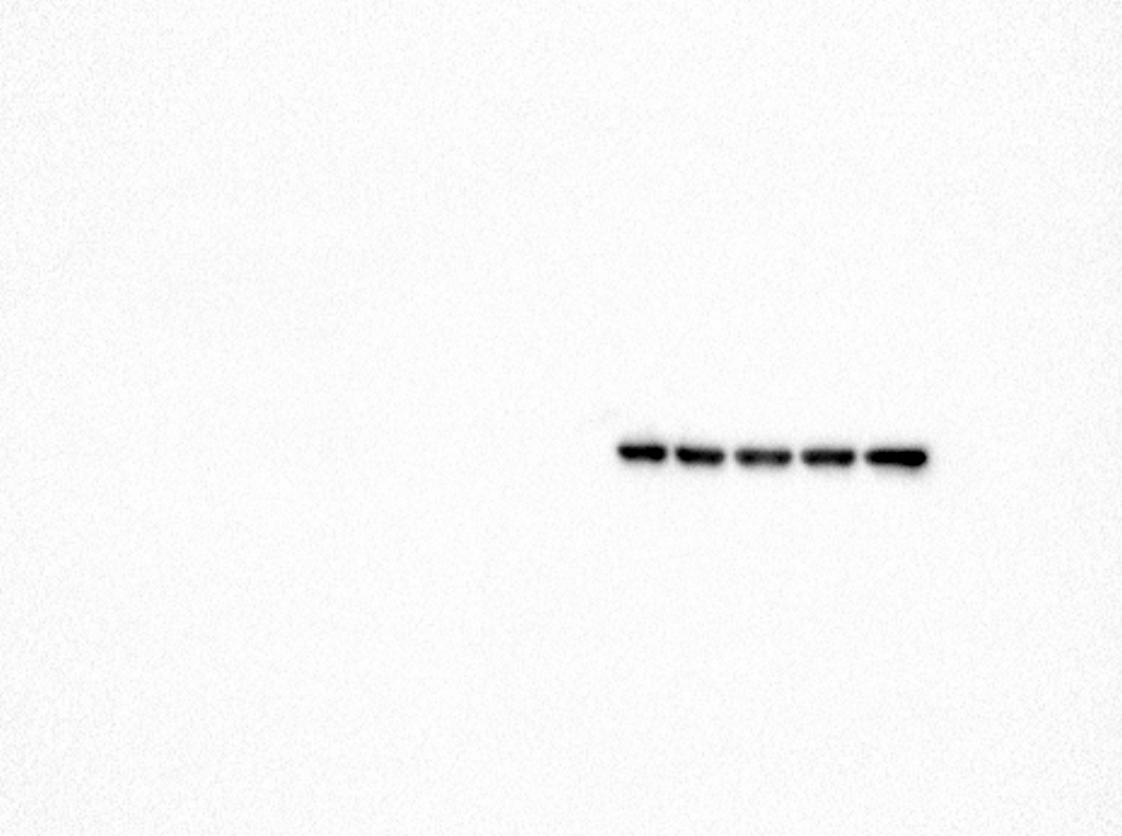

Supplement: Supplementary file 1 — Additional file 1. Data and analysis of this study. [file 12894_2022_1087_MOESM1_ESM.zip › Supplementary/GAPDH 3.tif]

Figure Legends

Image of IHC(Normal)

Figure S1-10 The expression of AKT1 in normal tissues.


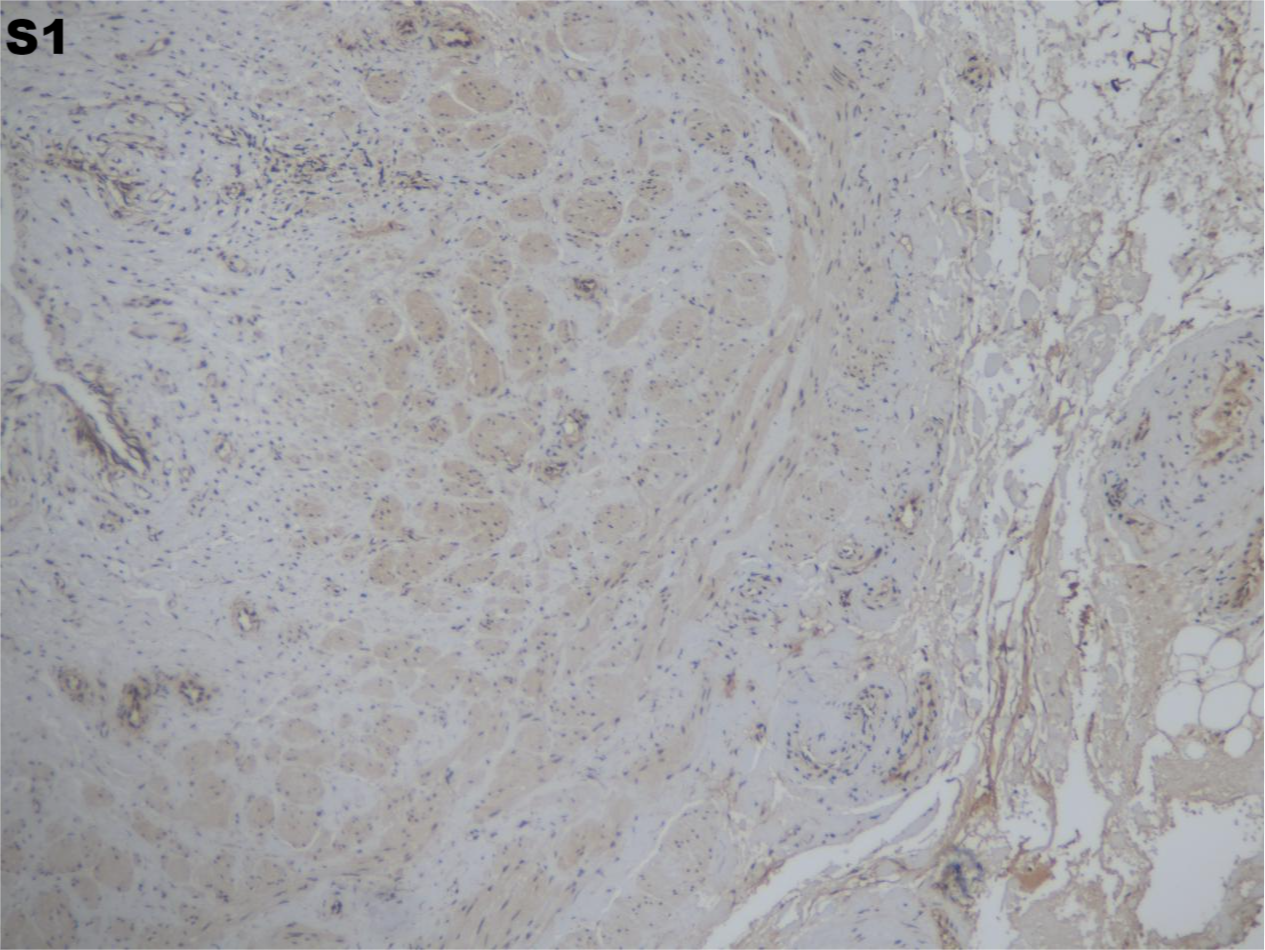


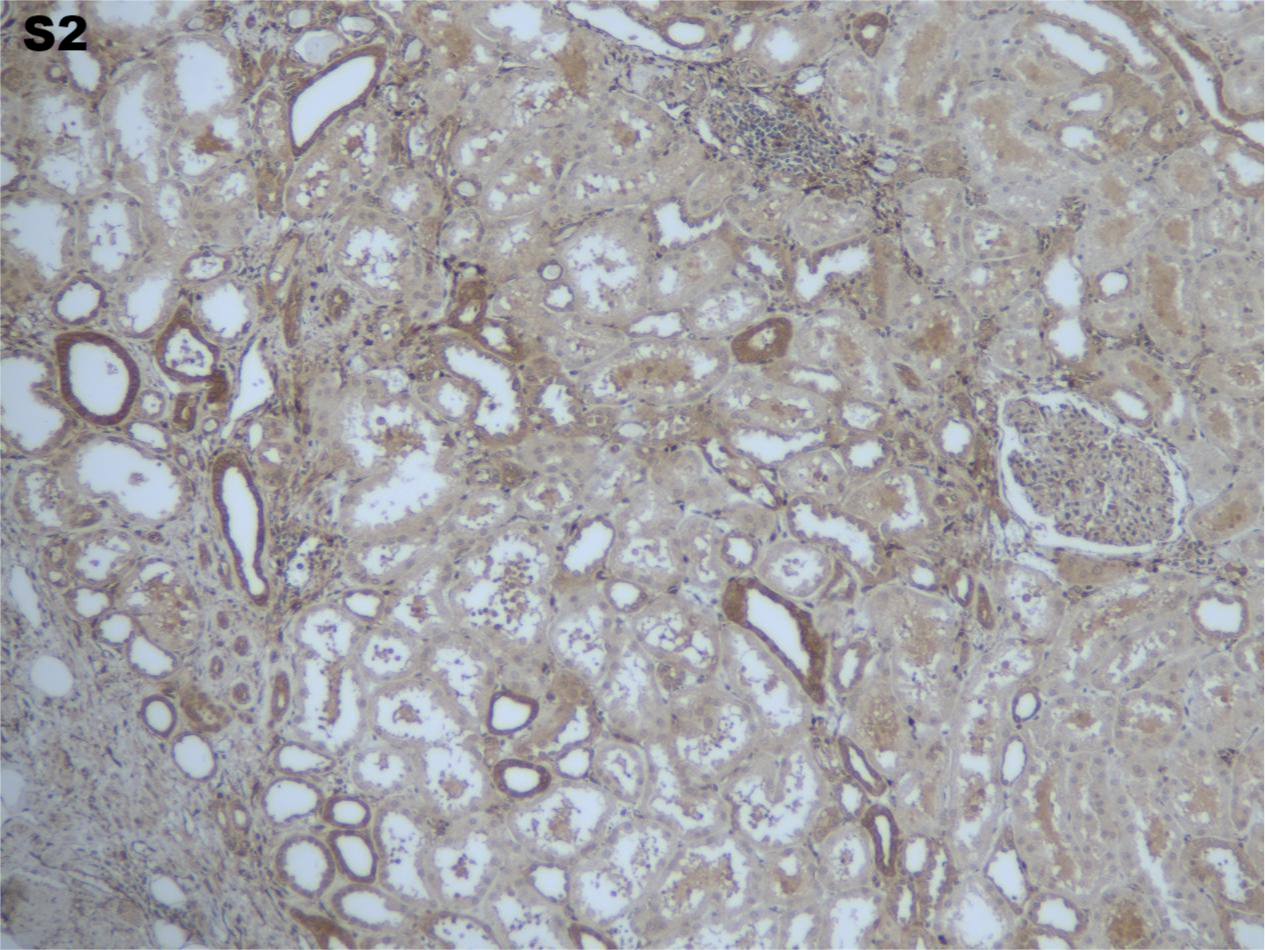


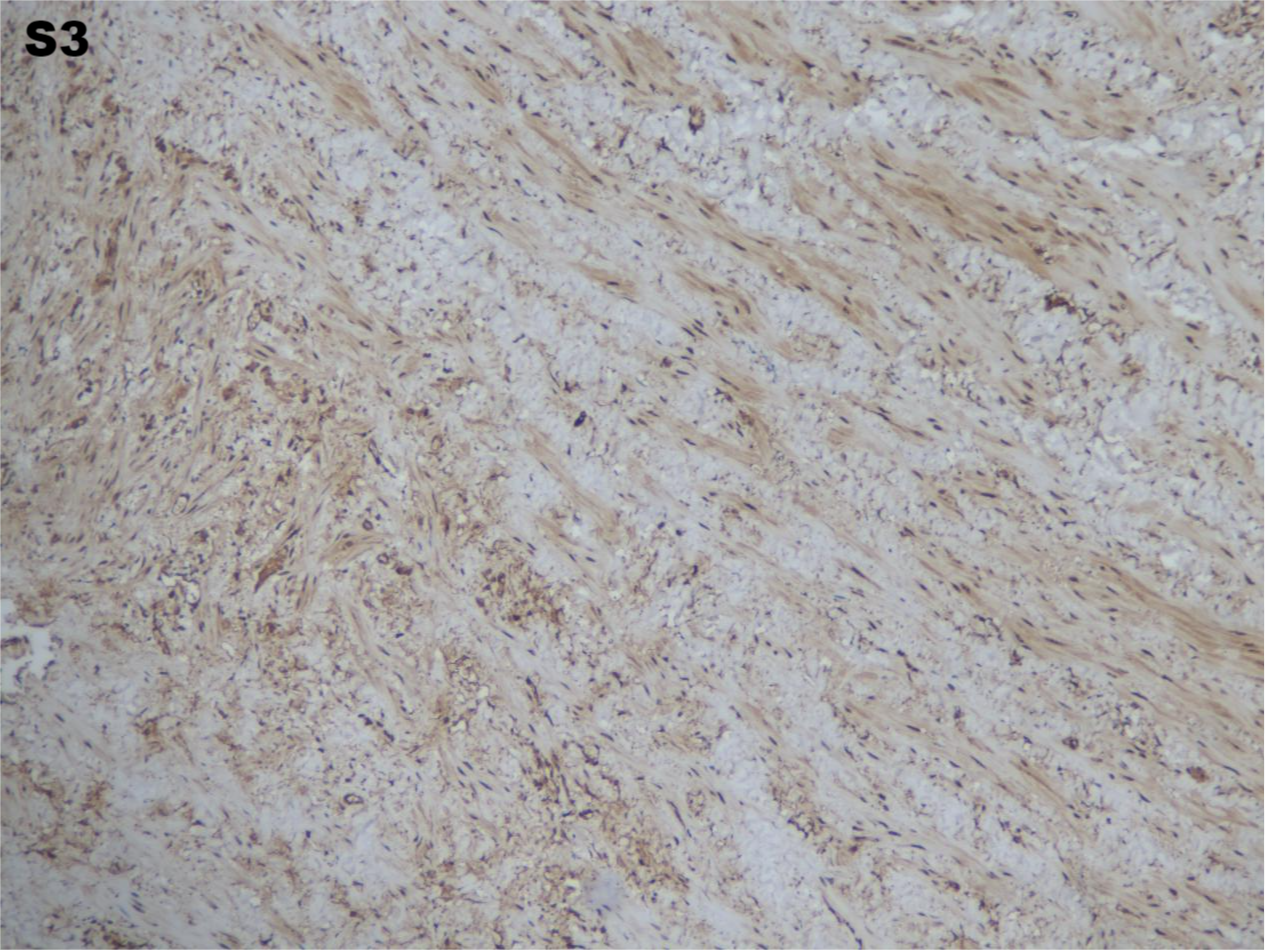


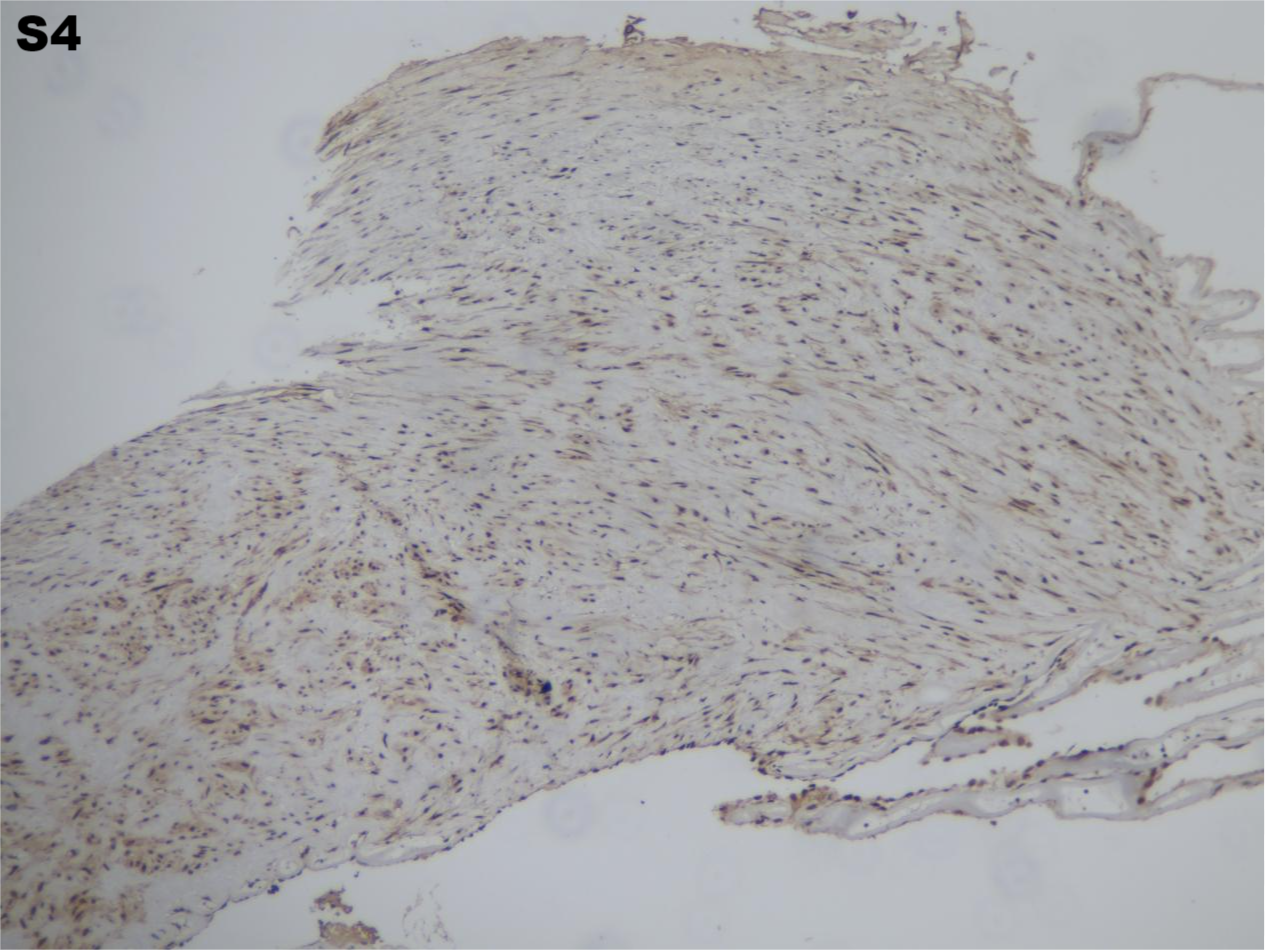


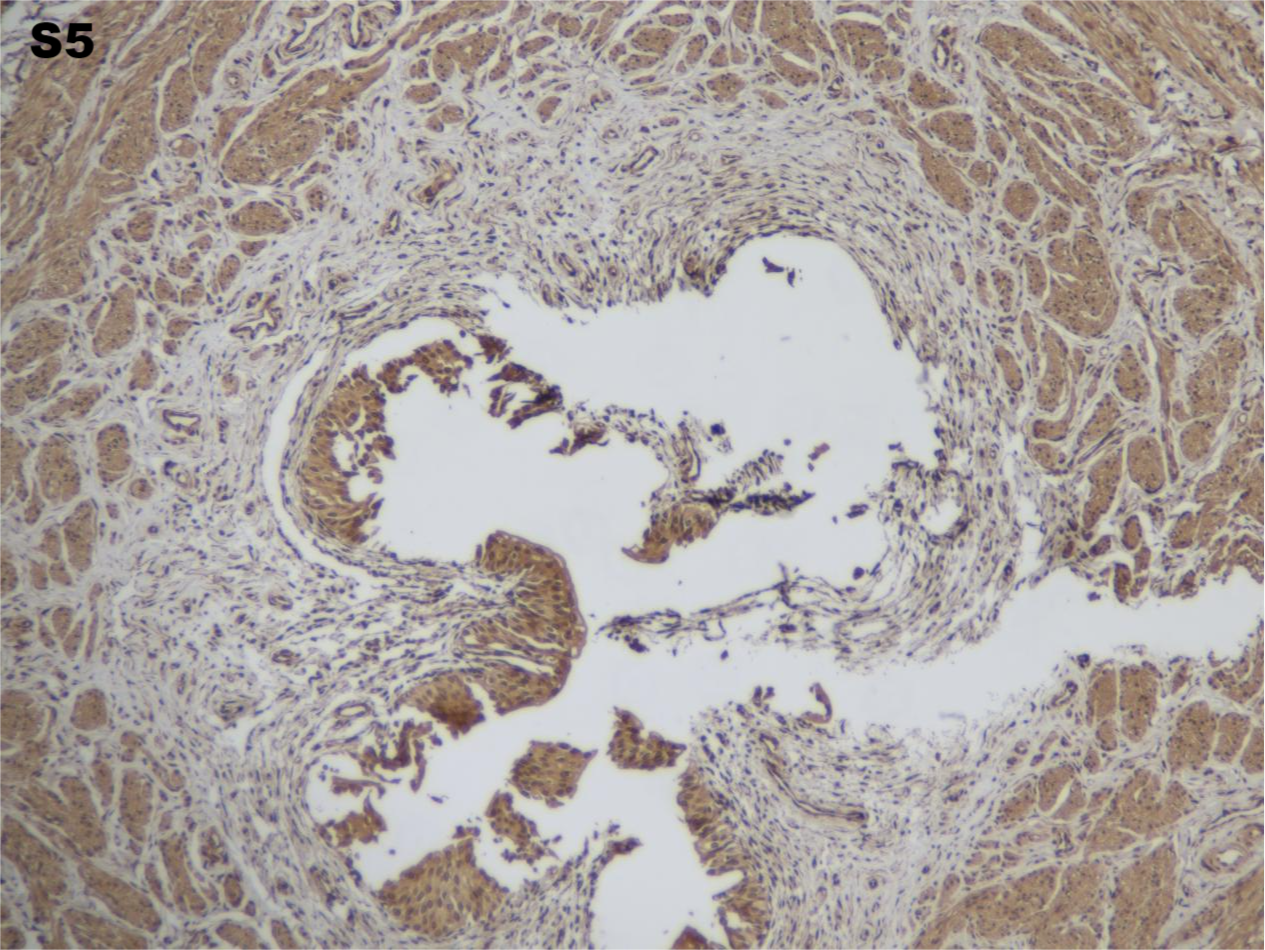


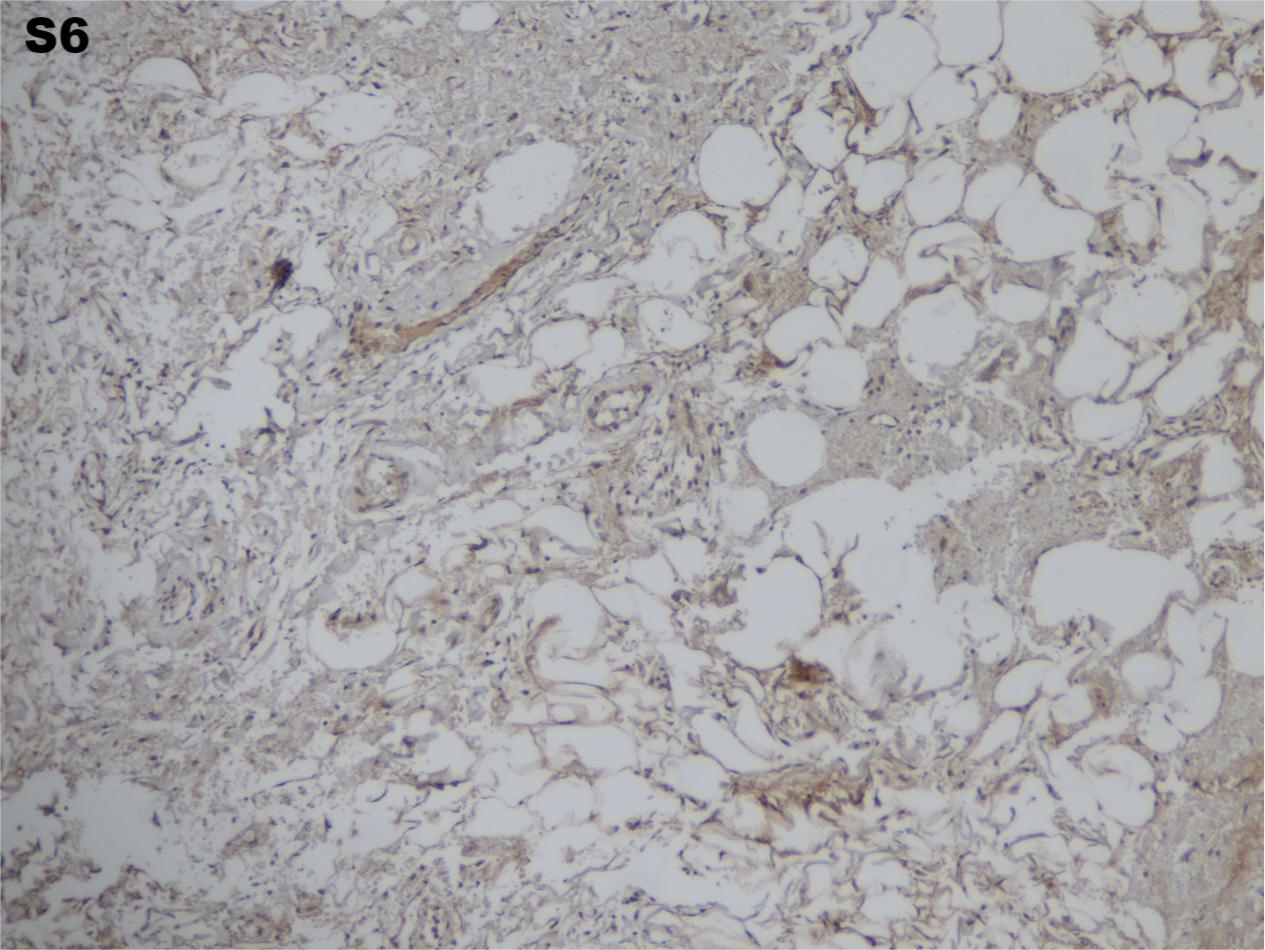


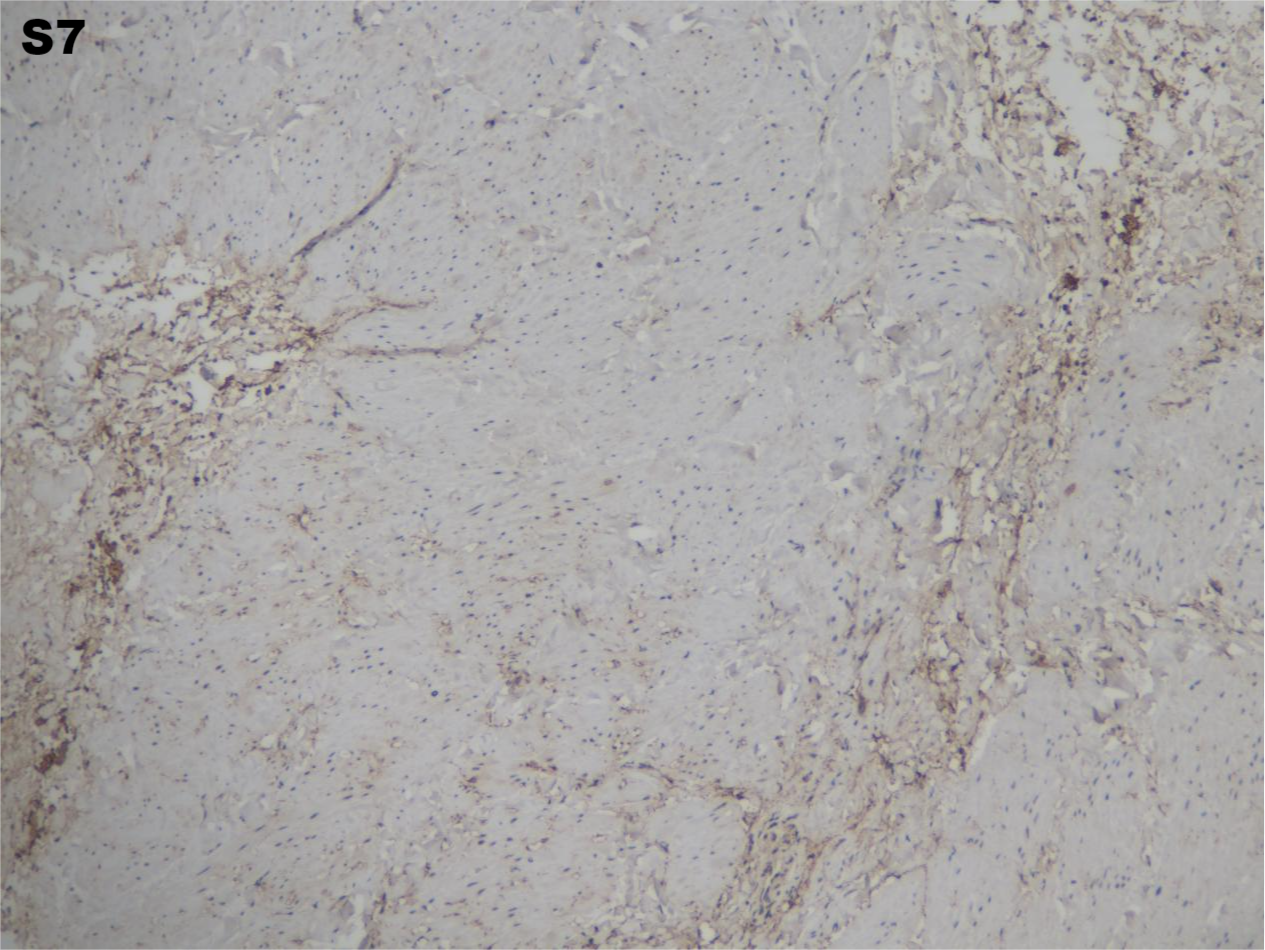


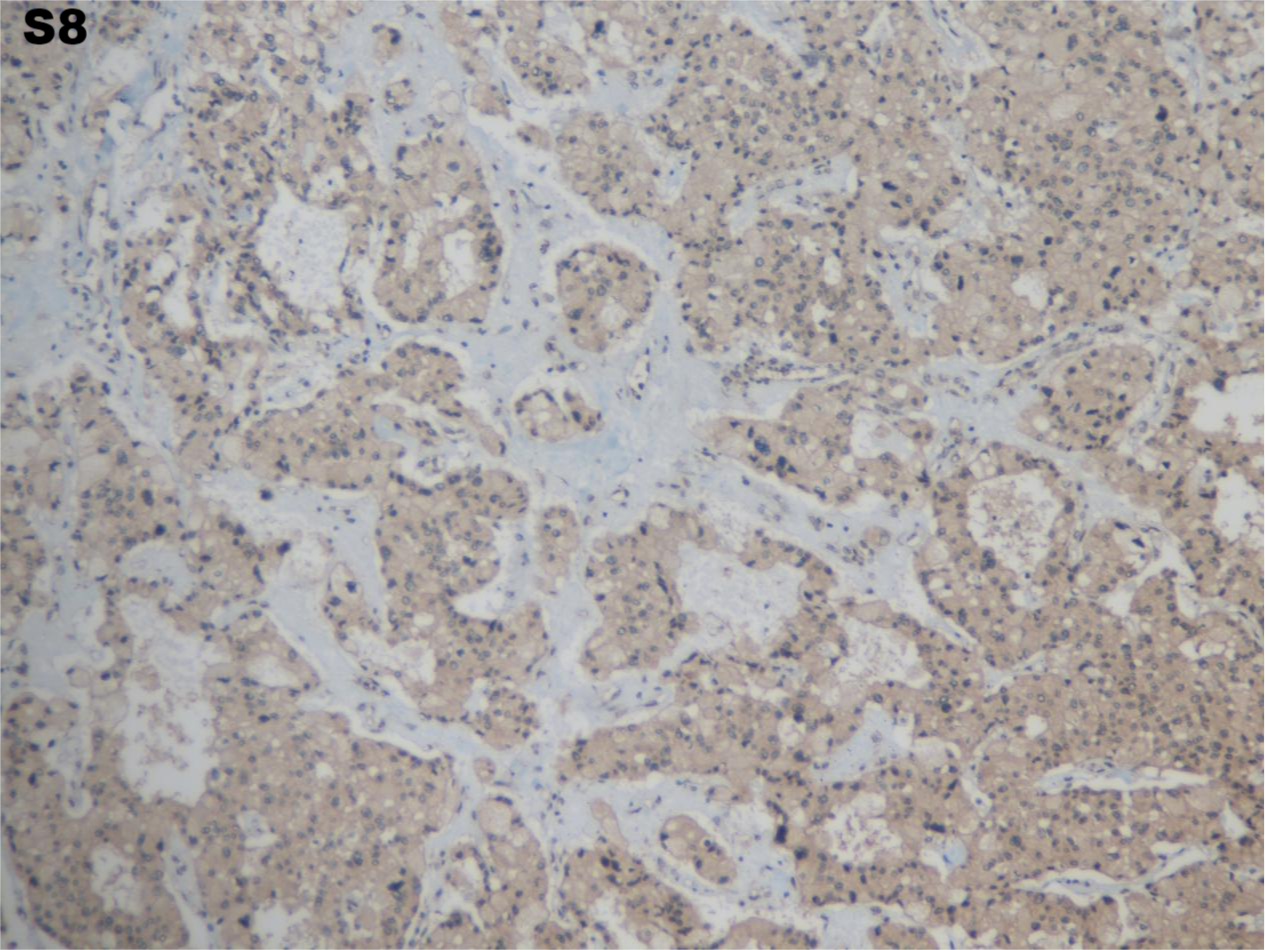


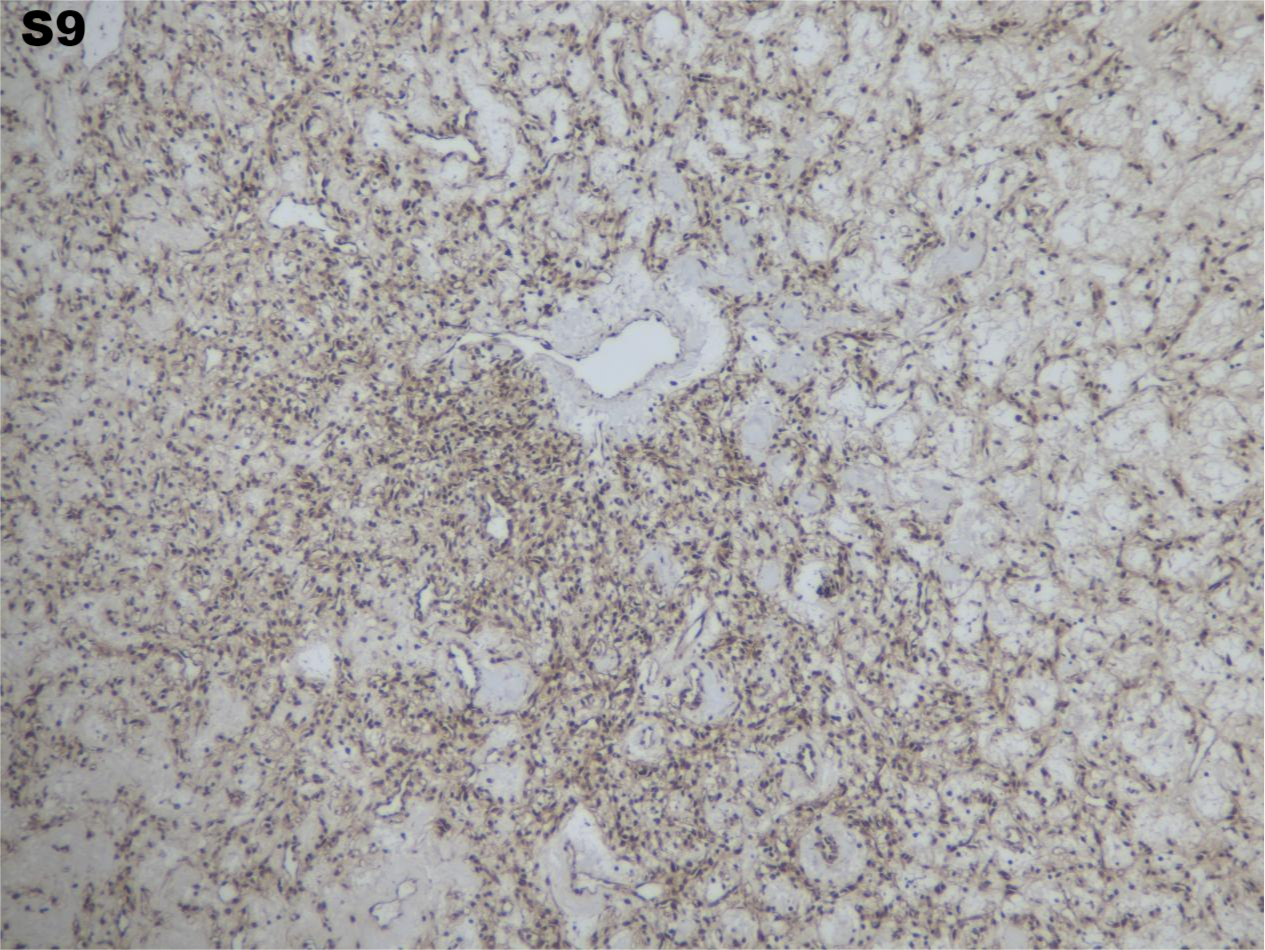


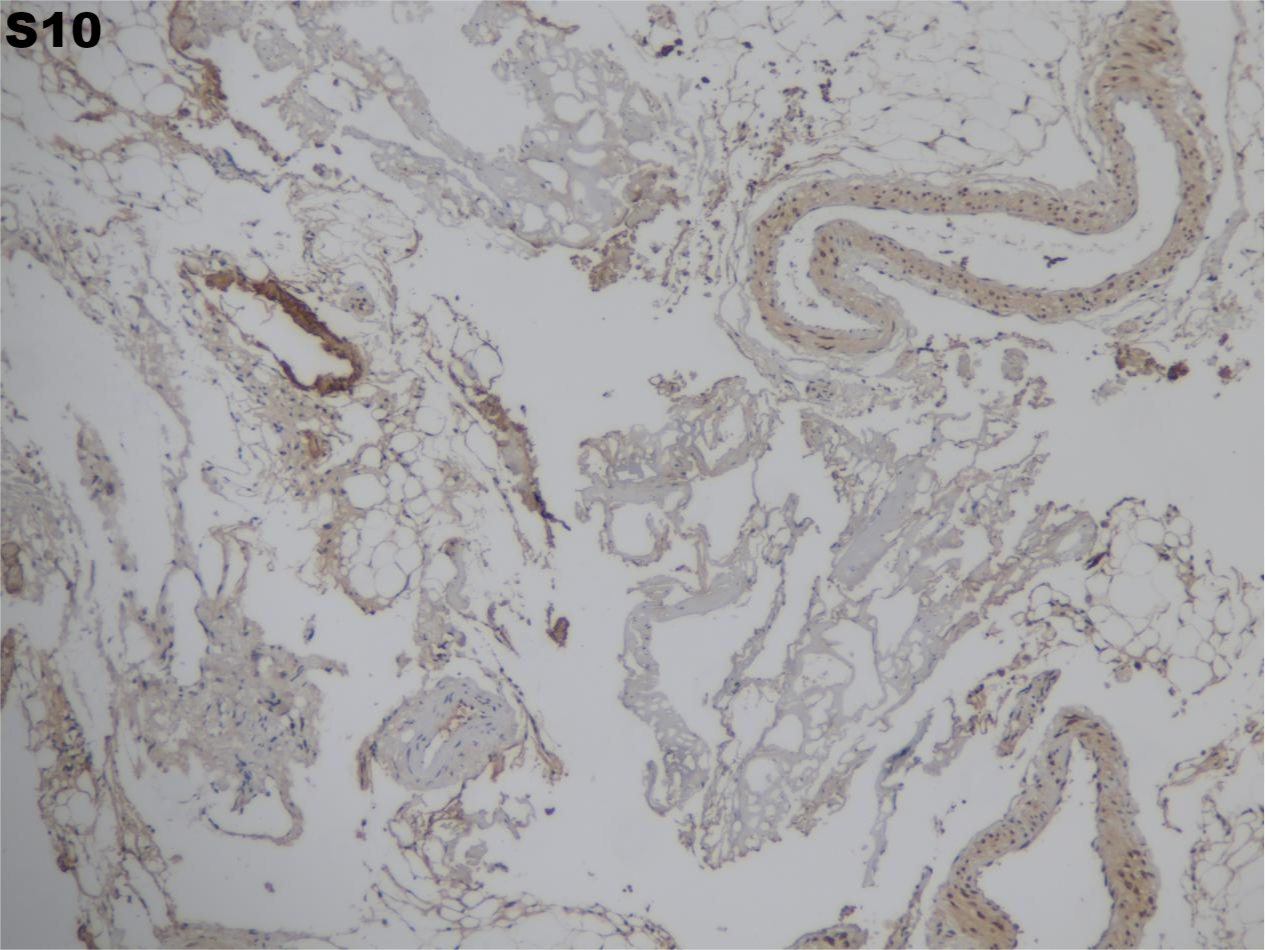

Supplement: Supplementary file 1 — Additional file 1. Data and analysis of this study. [file 12894_2022_1087_MOESM1_ESM.zip › Supplementary/Image of IHC(N)(1).docx]

Figure Legends

Image of IHC(Normal)

Figure S11-21 The expression of AKT1 in normal tissues.


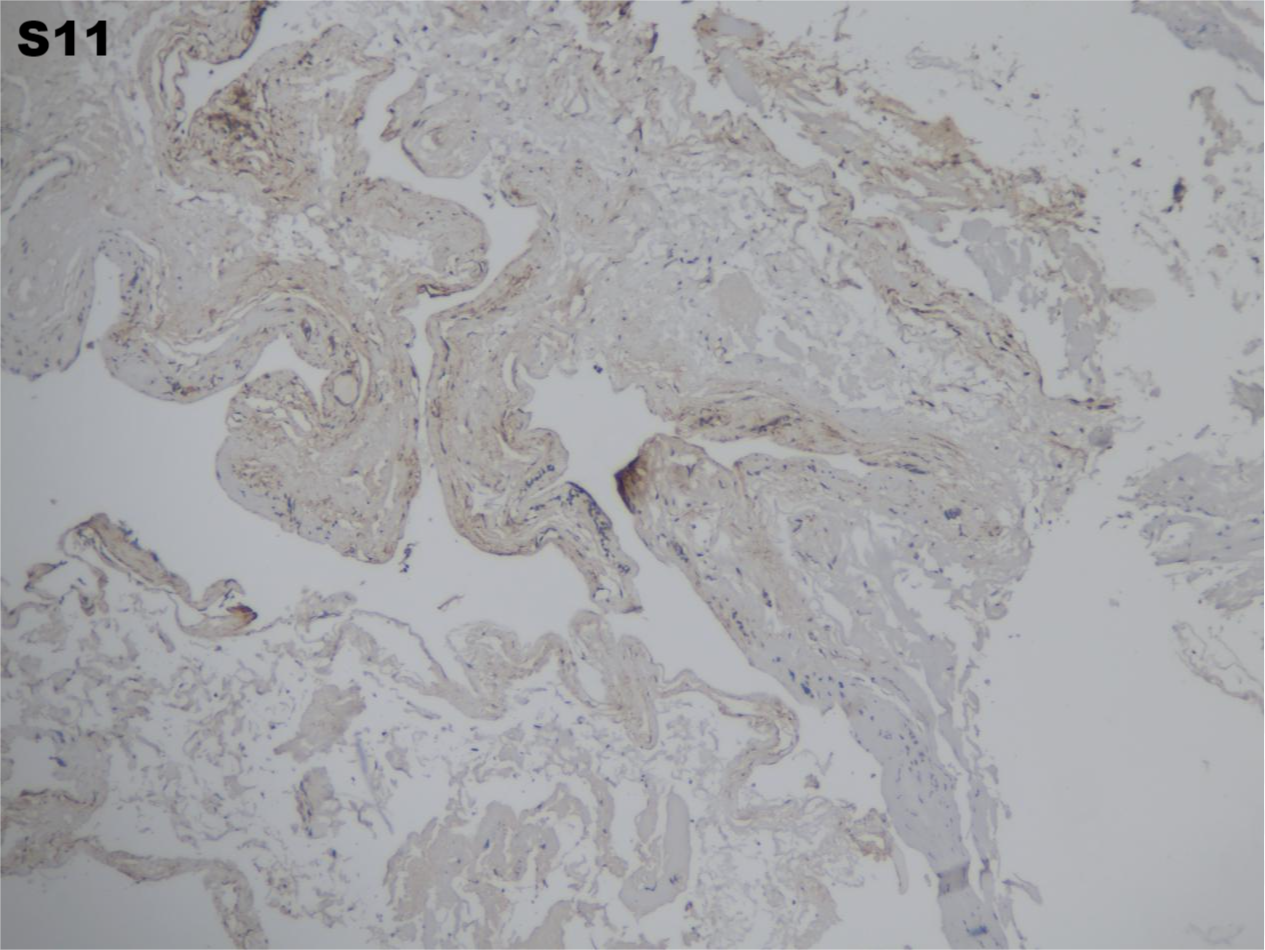


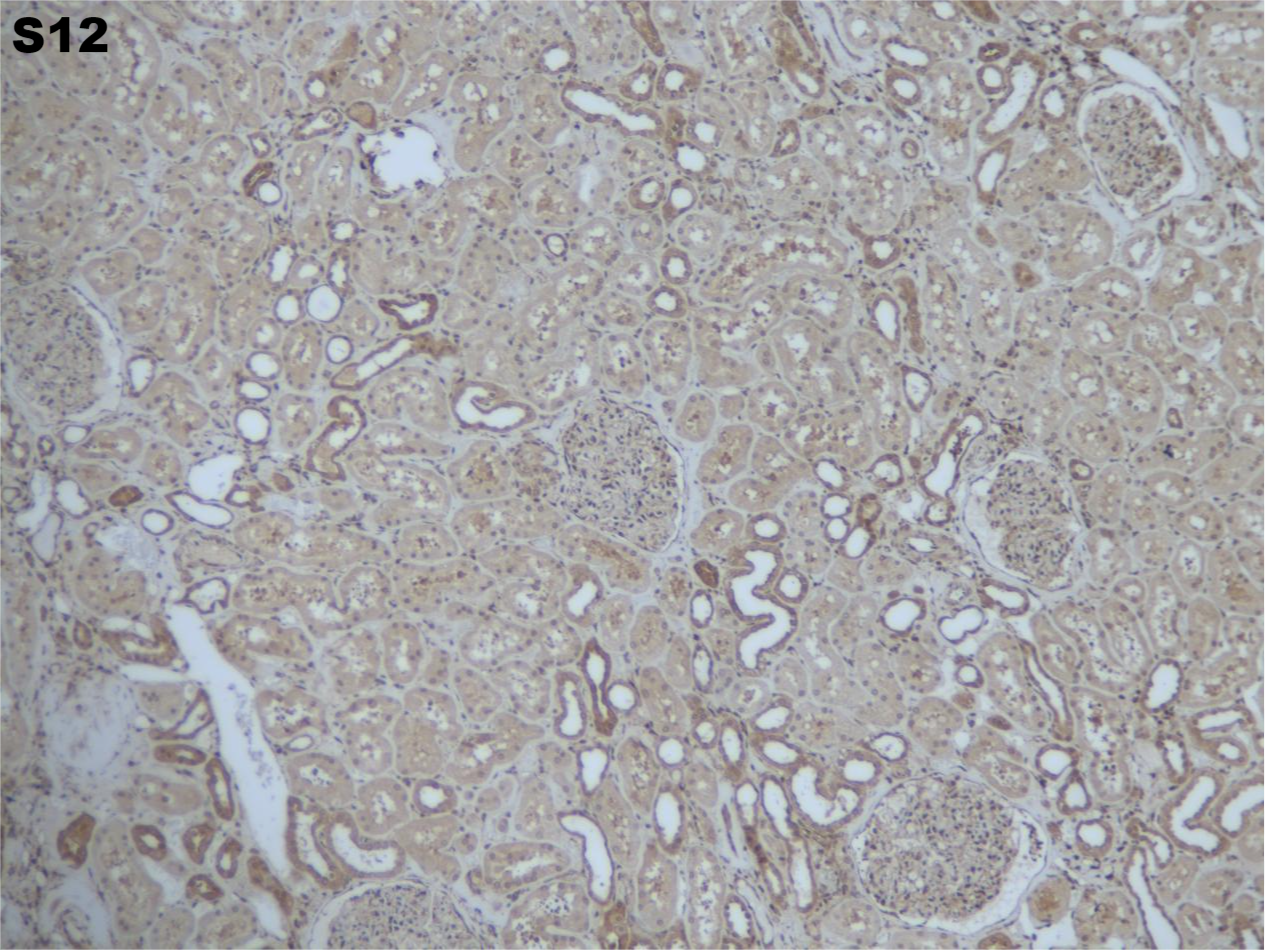


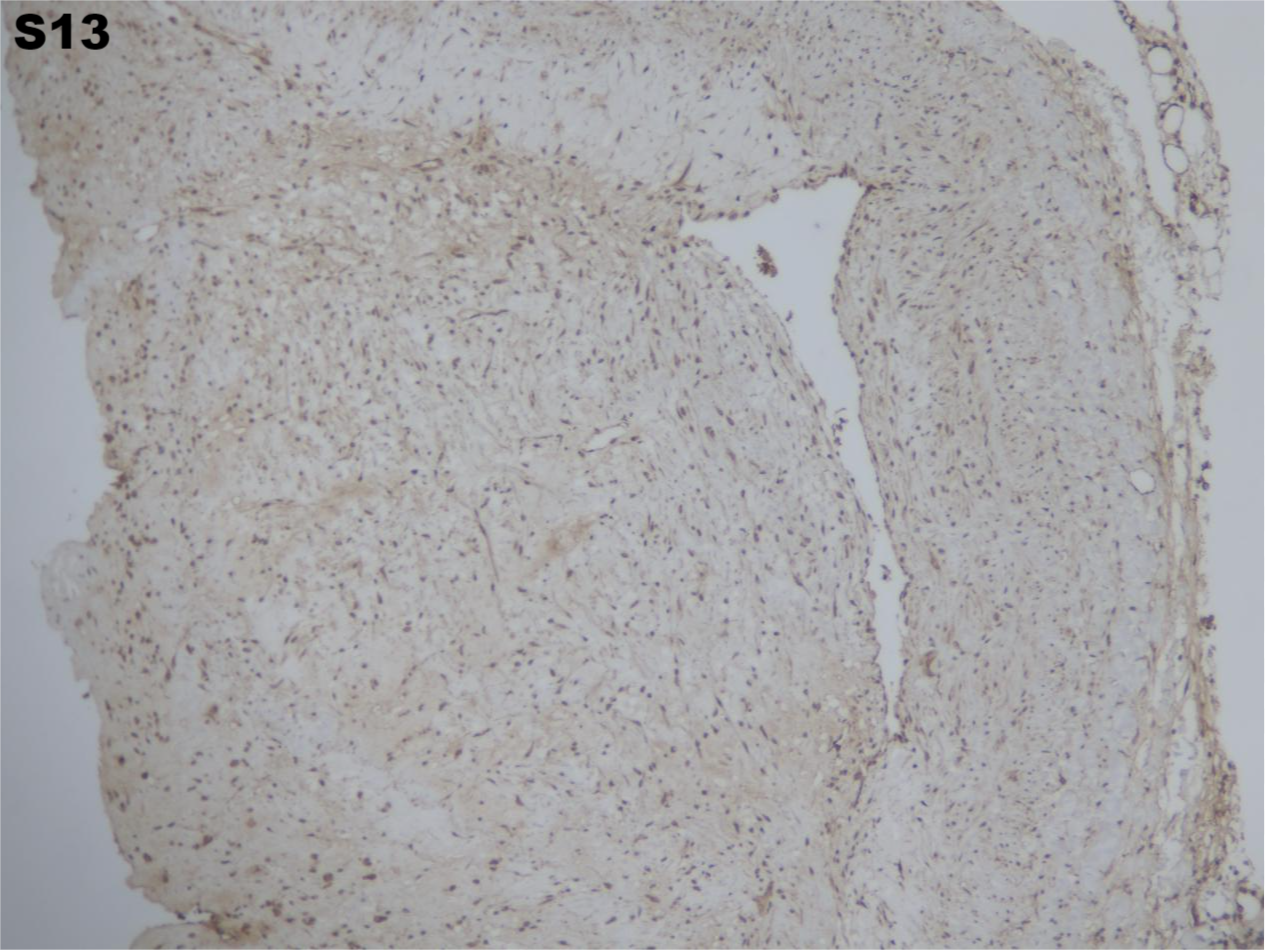


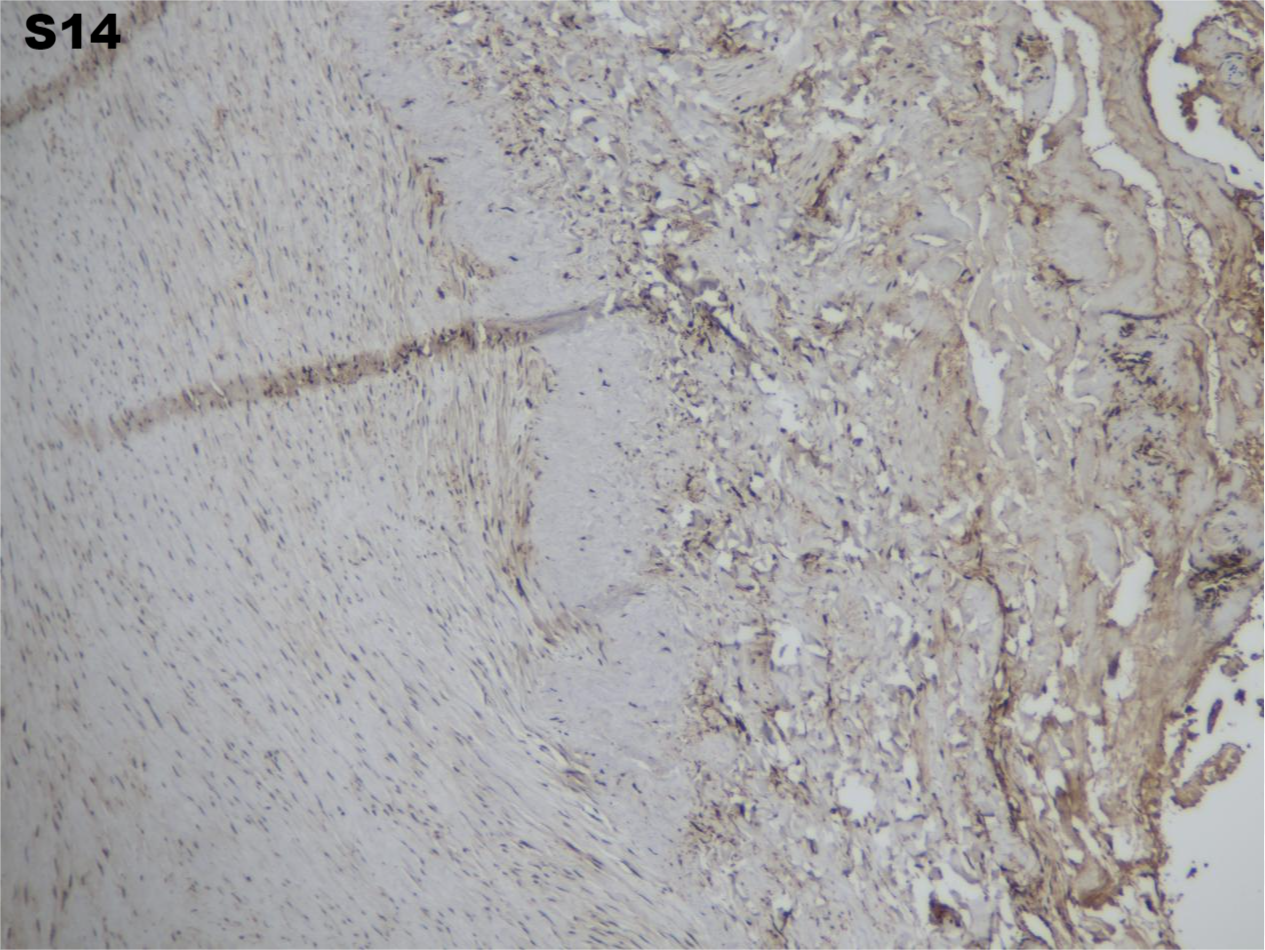


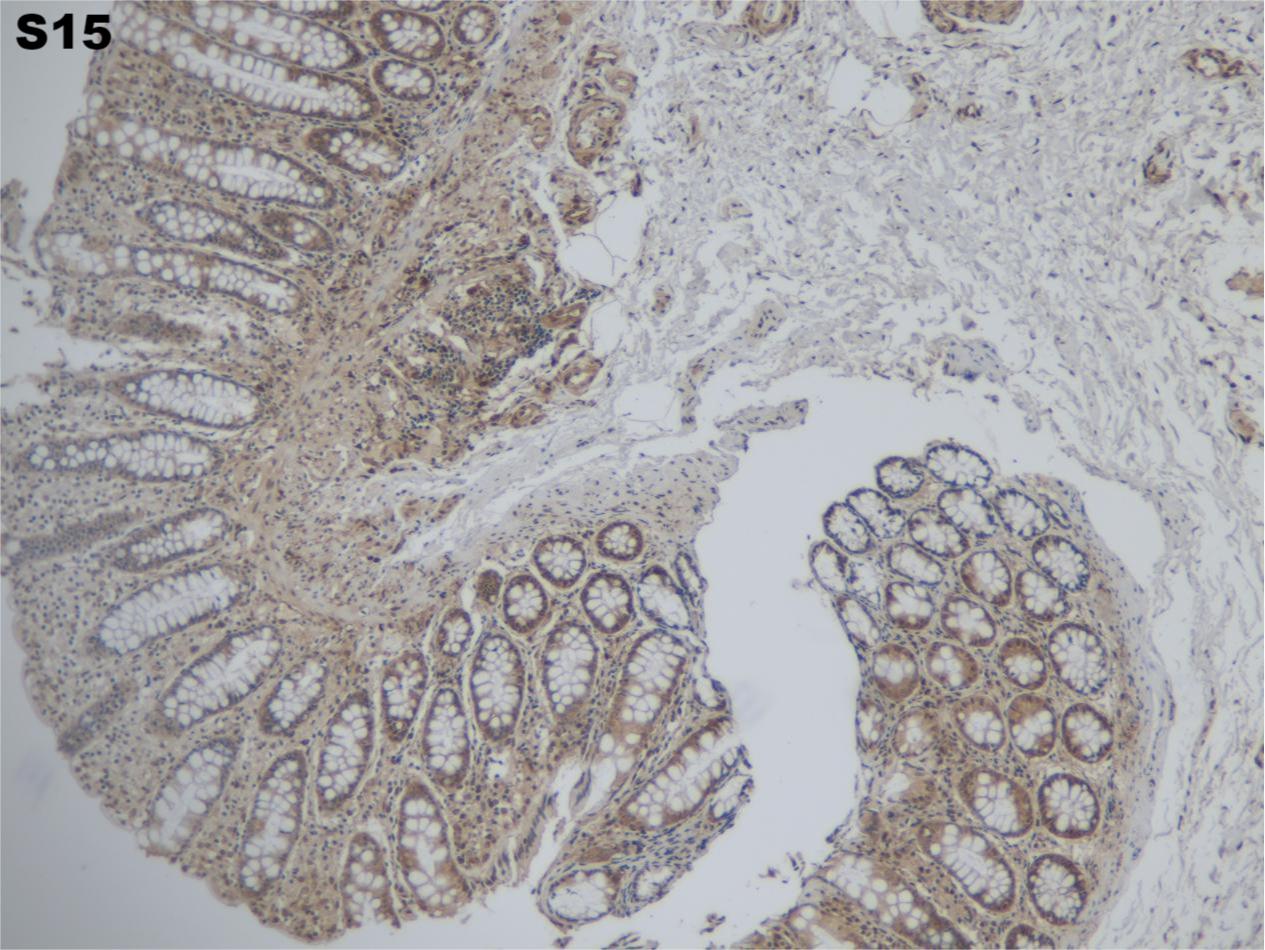


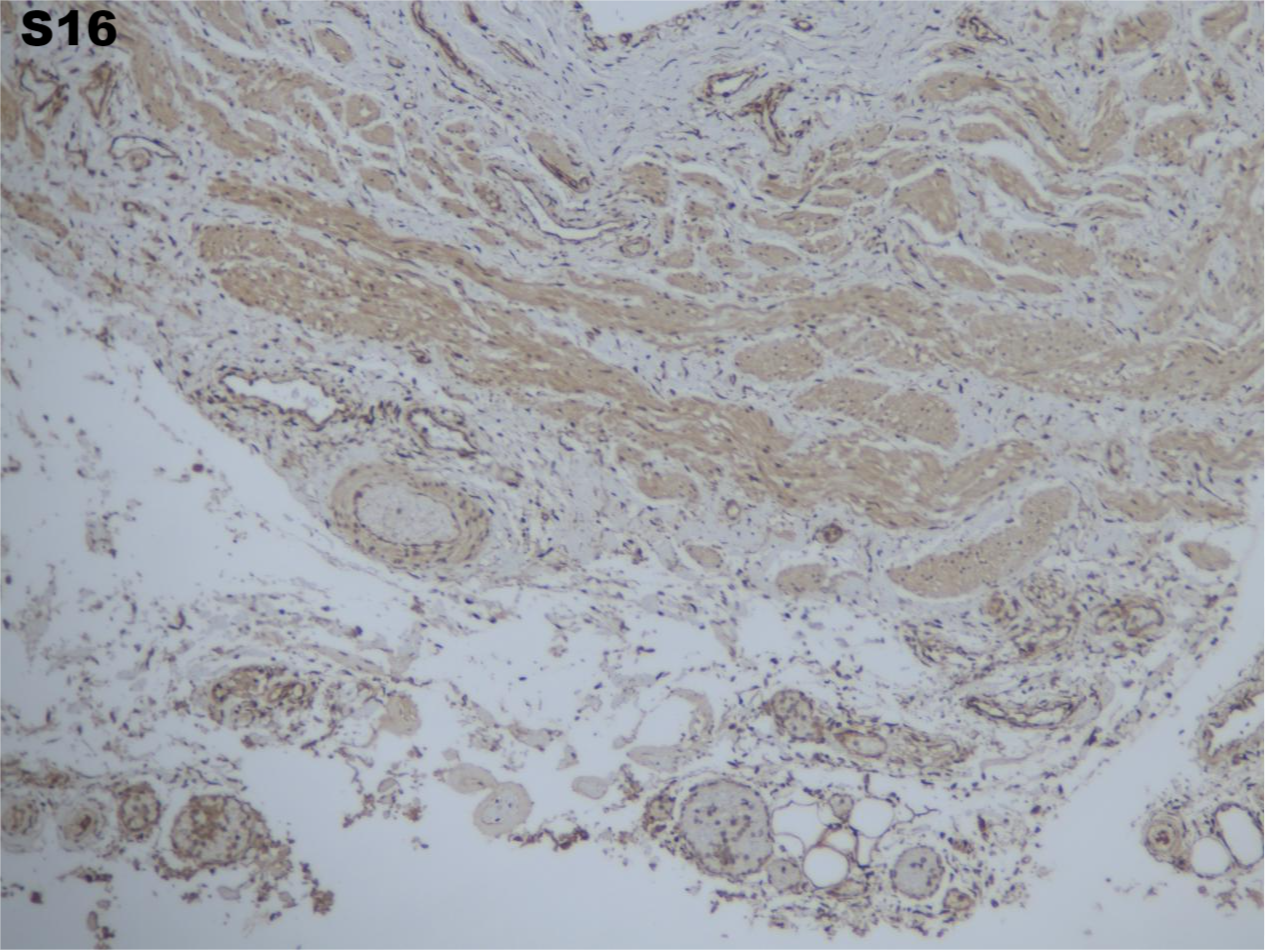


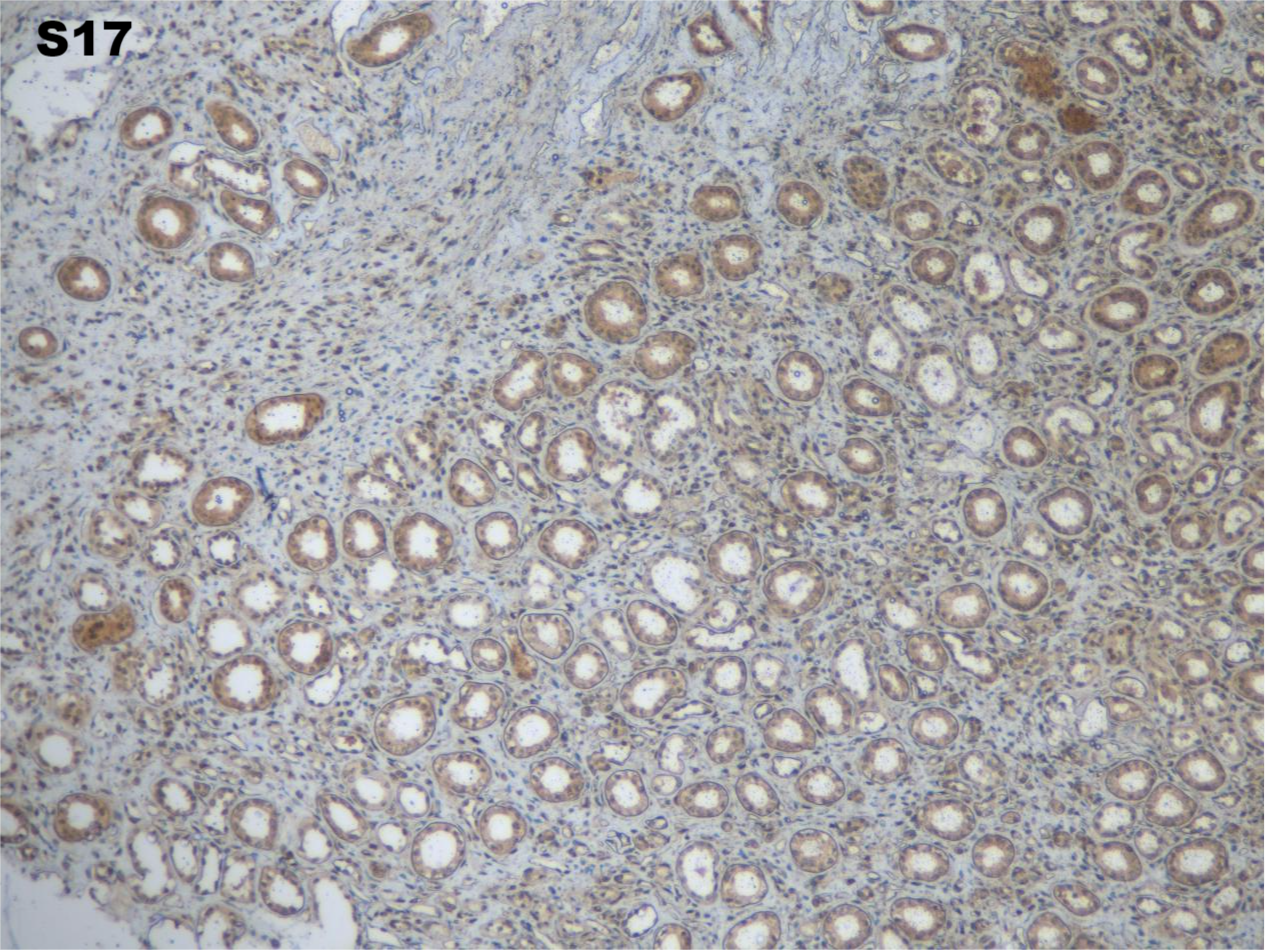


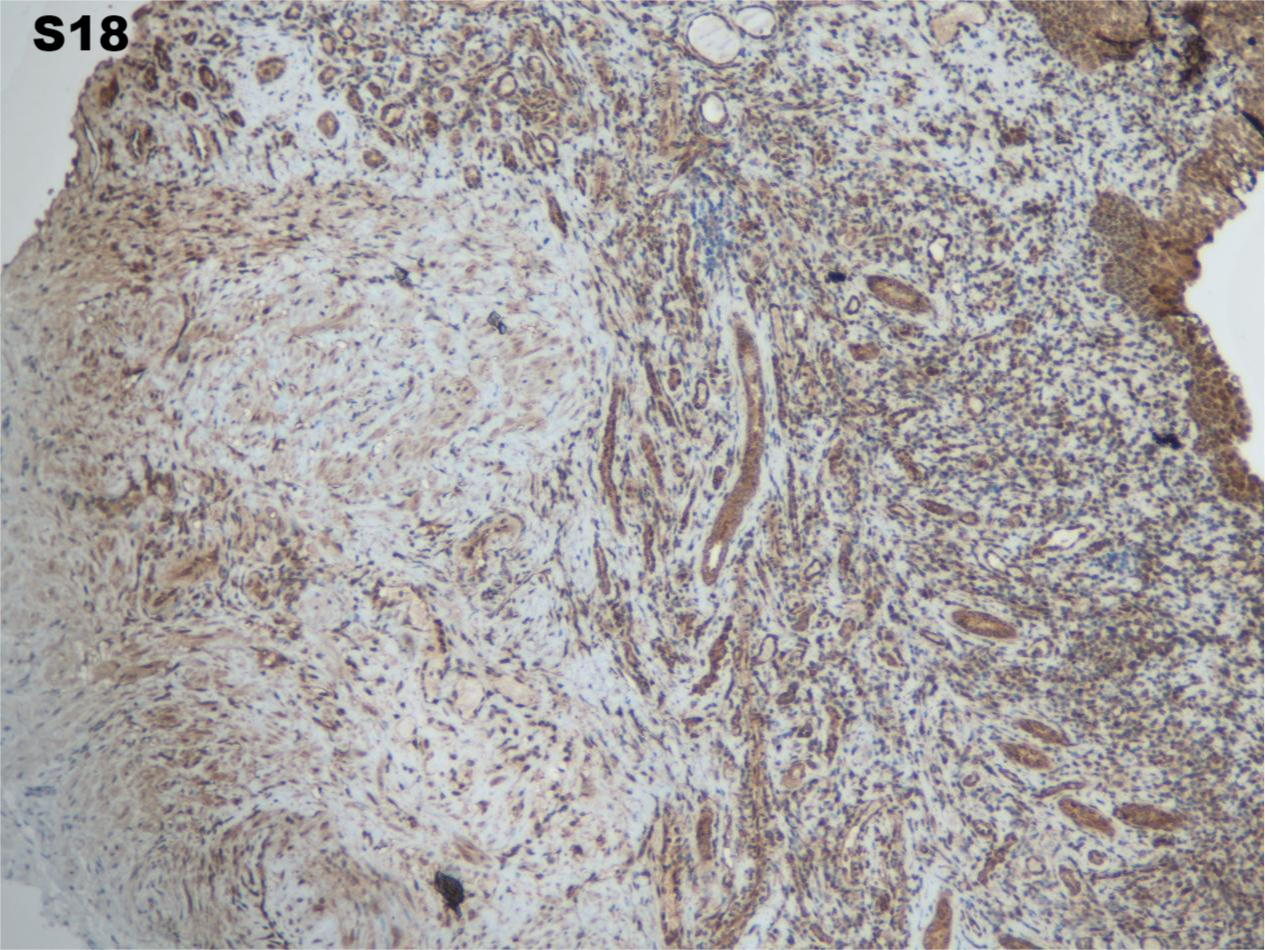


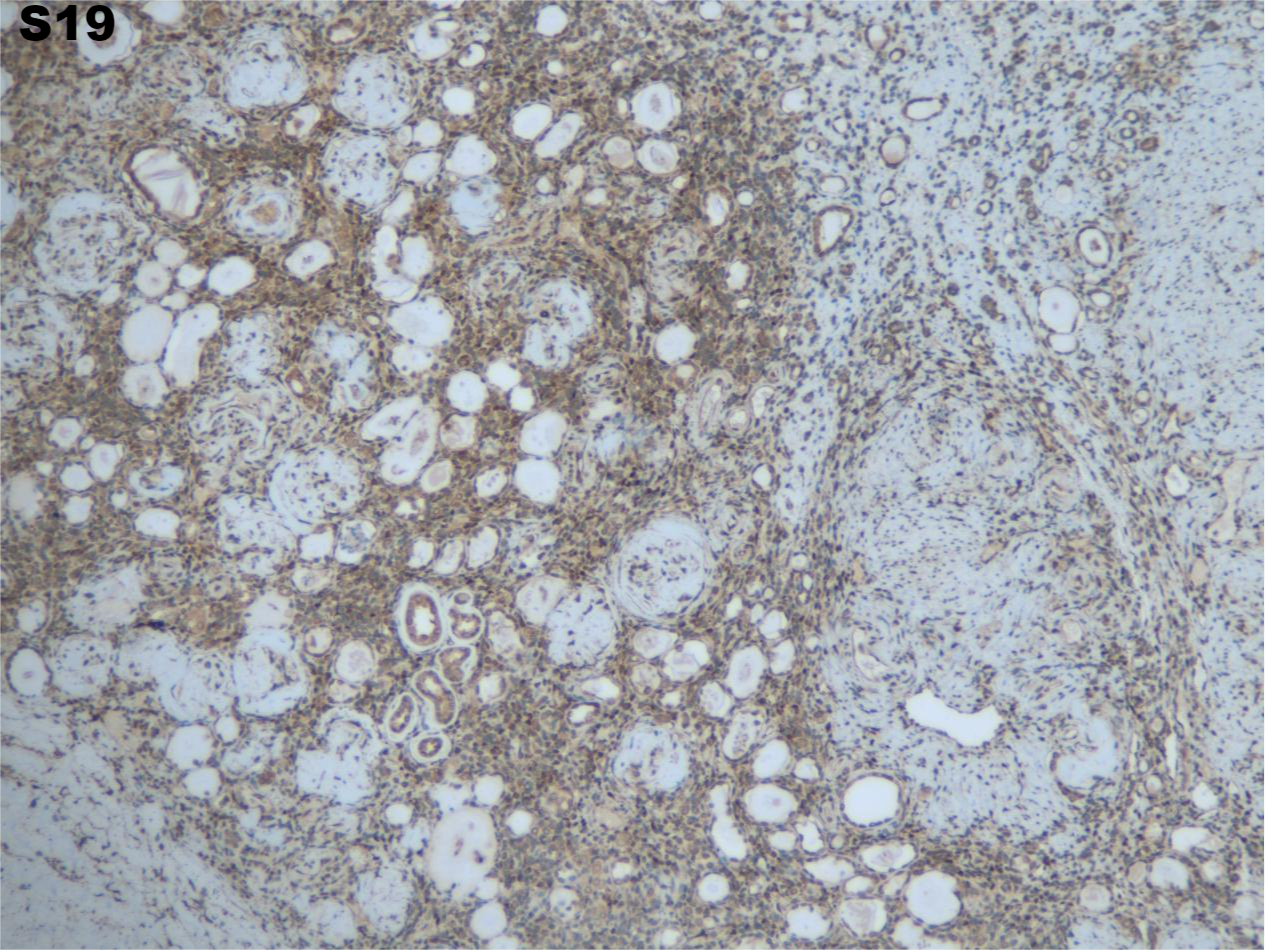


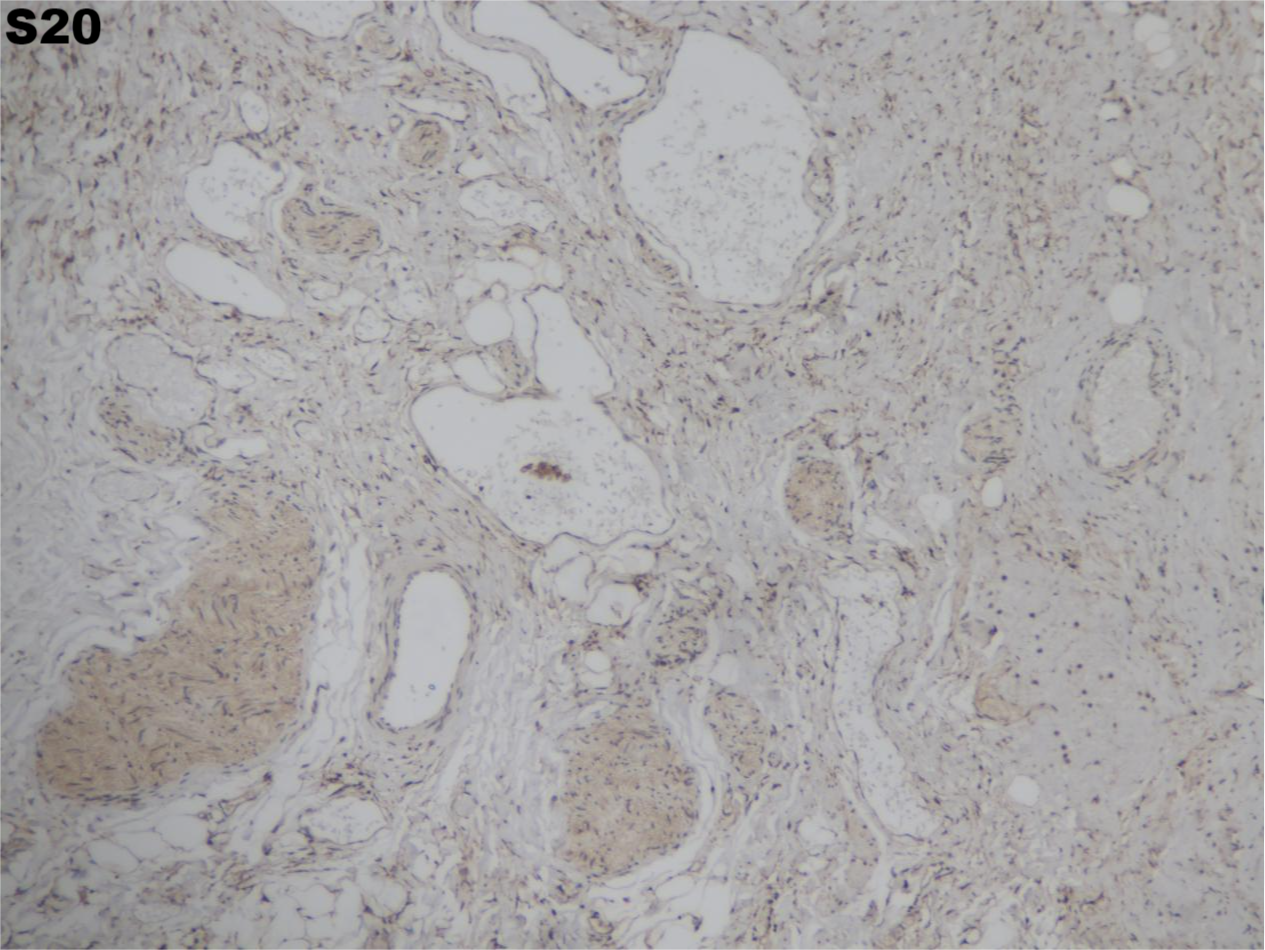


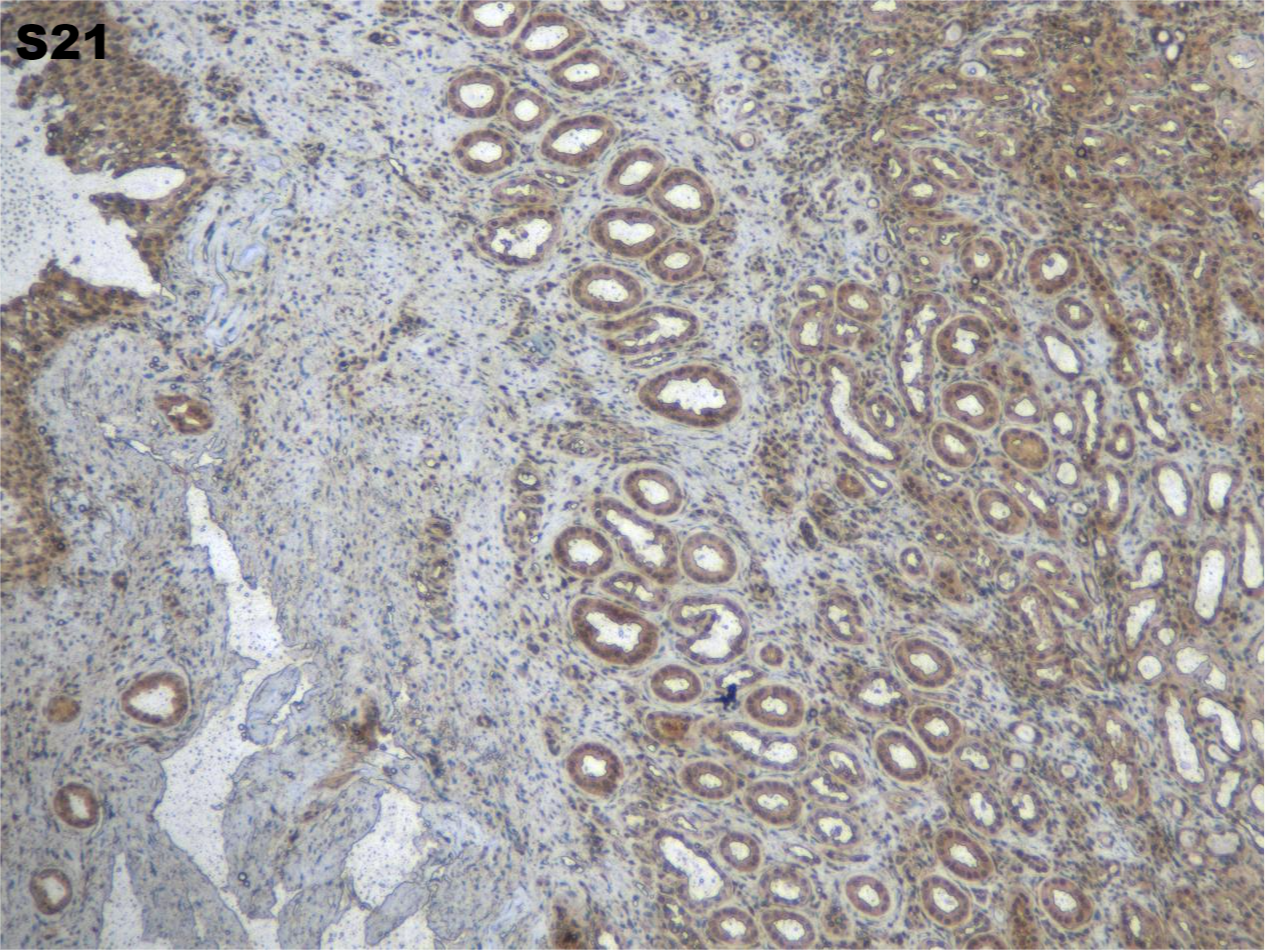

Supplement: Supplementary file 1 — Additional file 1. Data and analysis of this study. [file 12894_2022_1087_MOESM1_ESM.zip › Supplementary/Image of IHC(N)(2).docx]

Figure Legends

Image of IHC(Tumor)

Figure S1-10 The expression of AKT1 in tumor tissues.


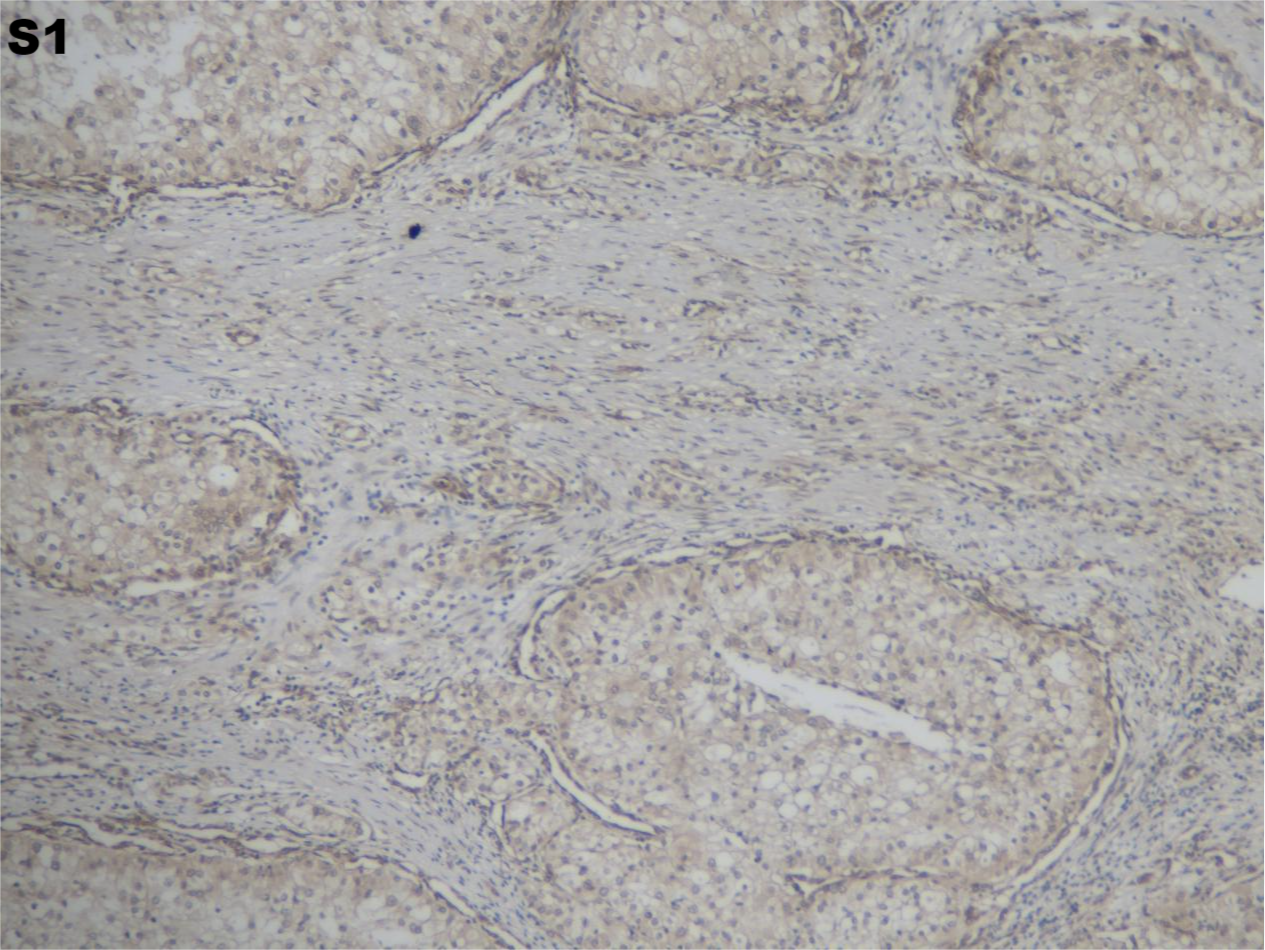


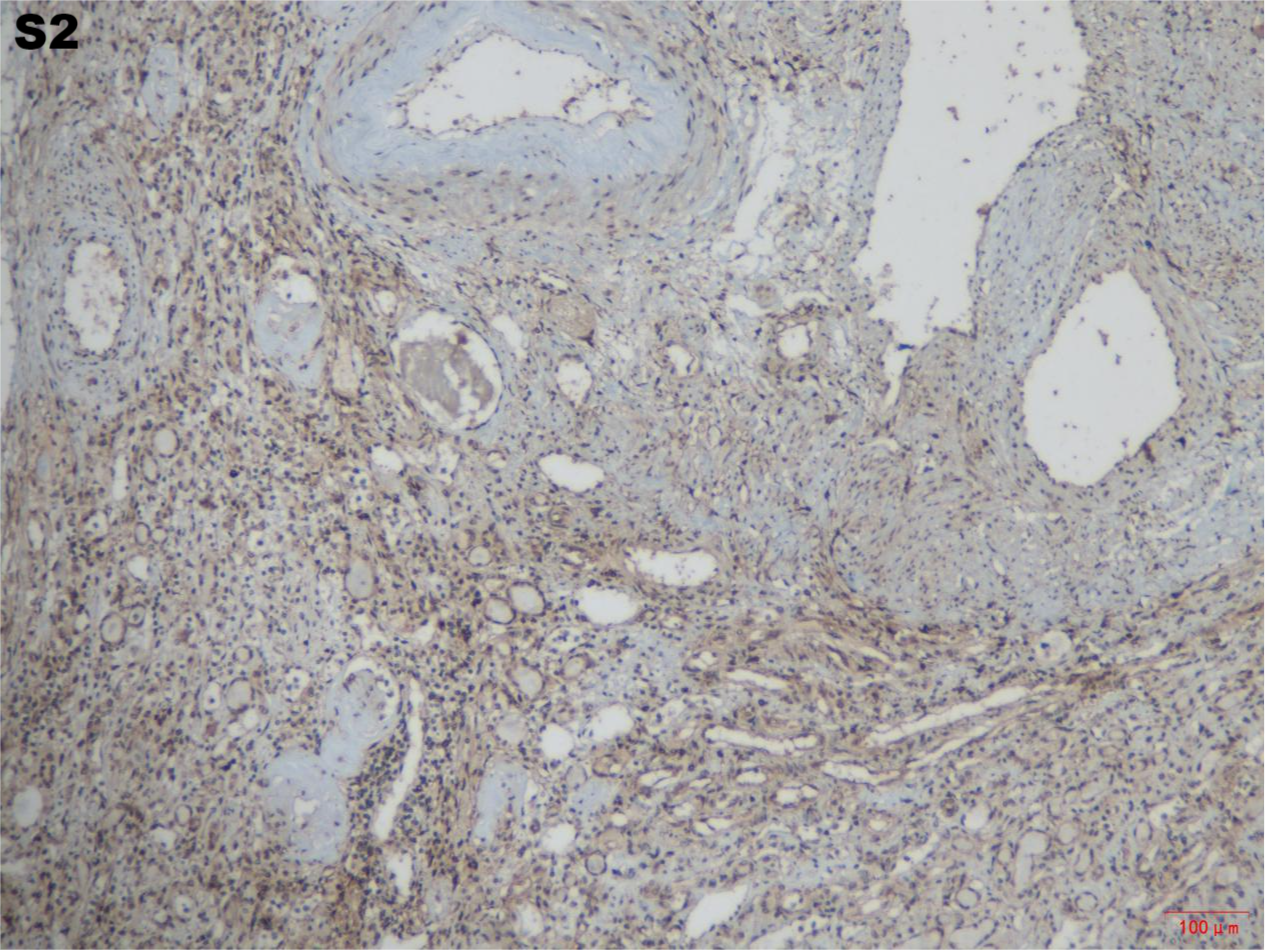


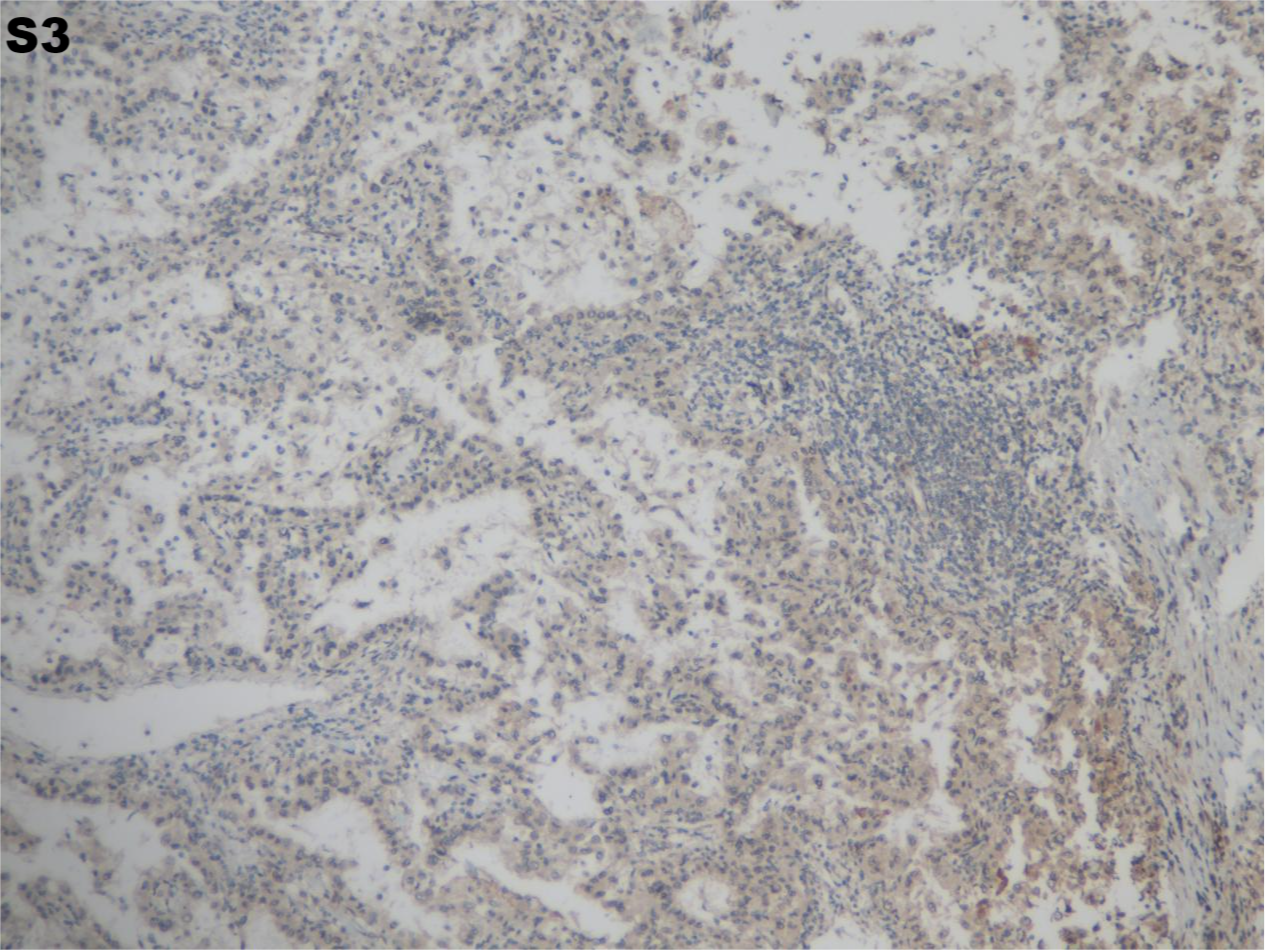


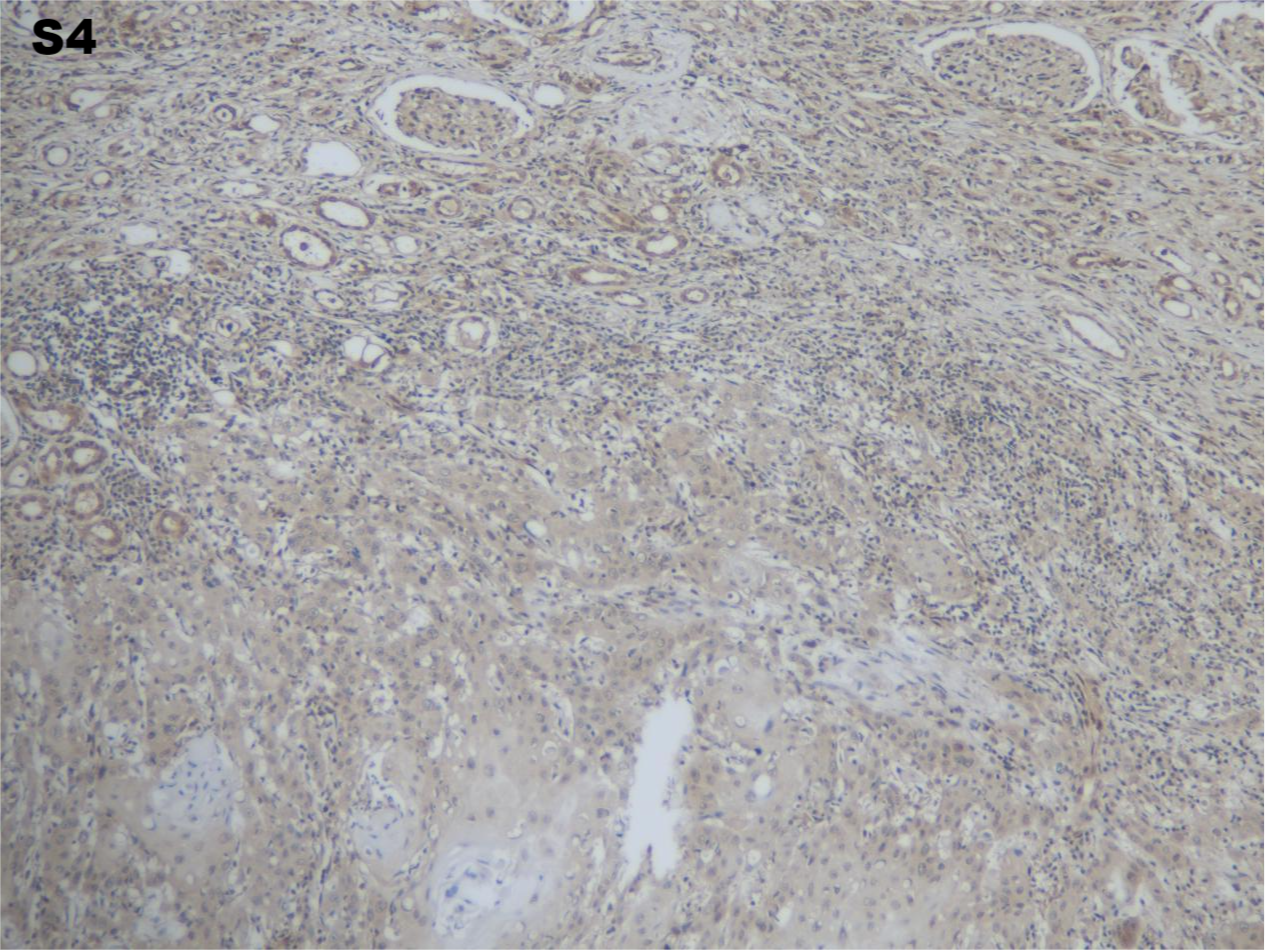


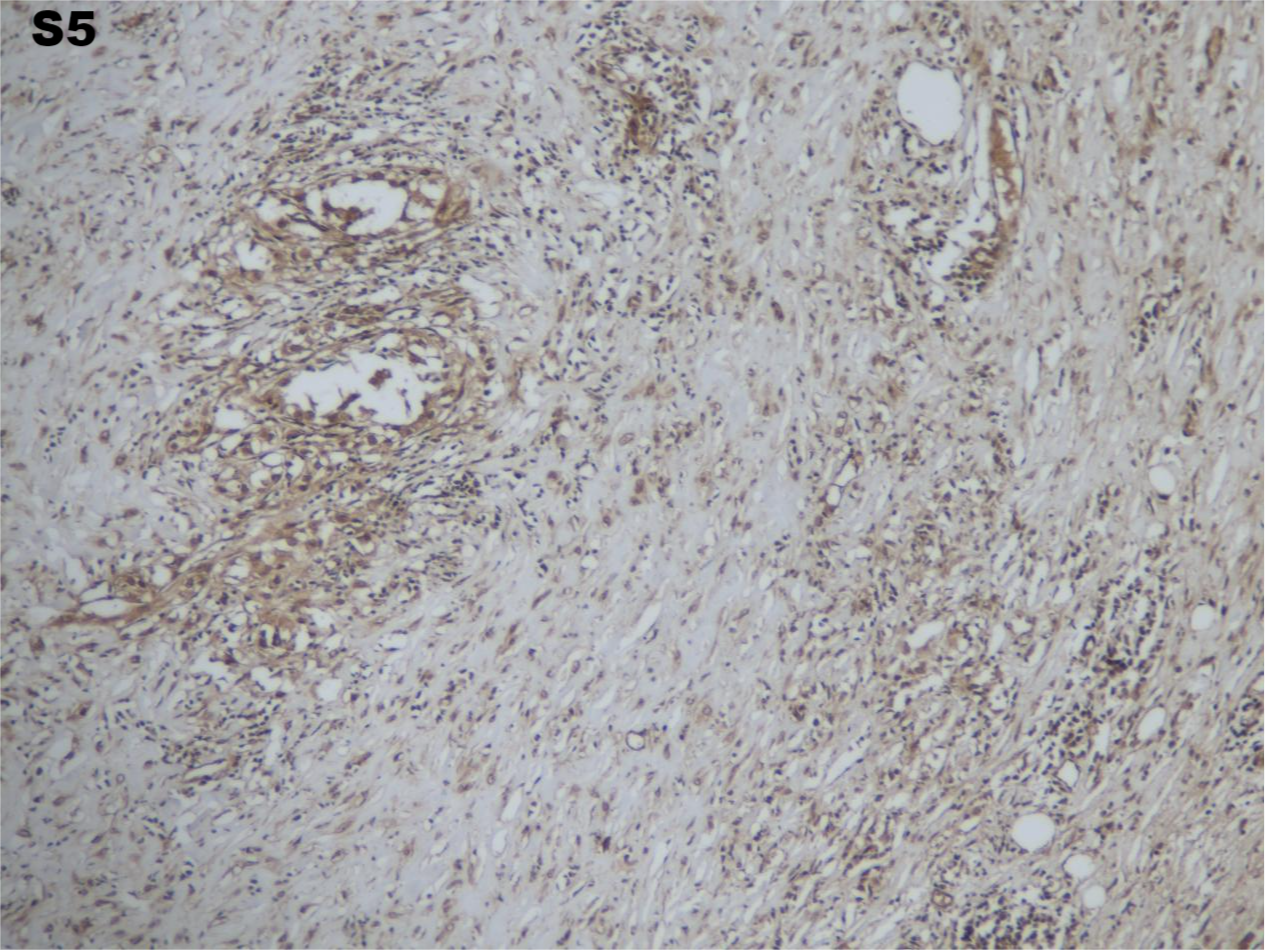


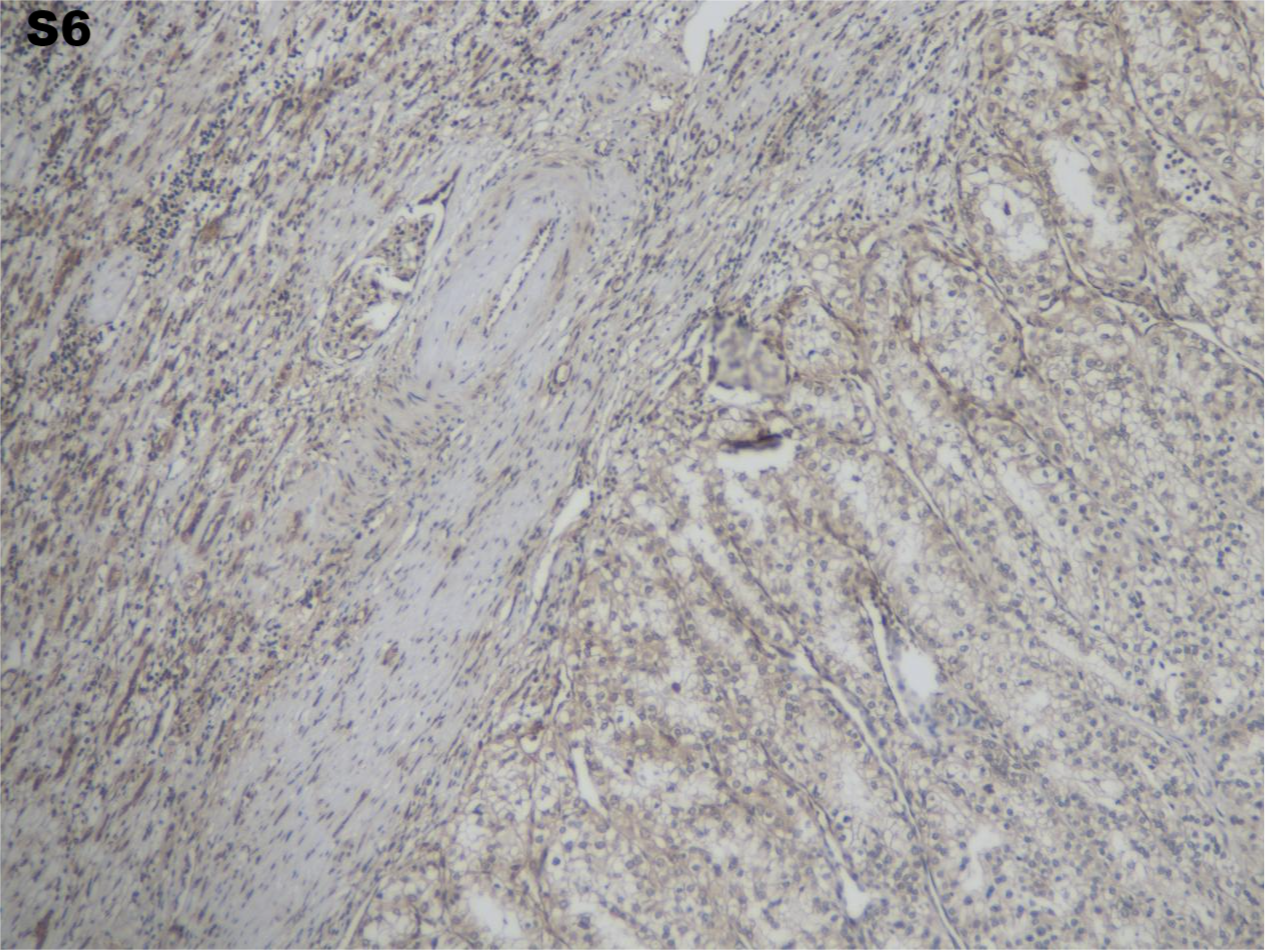


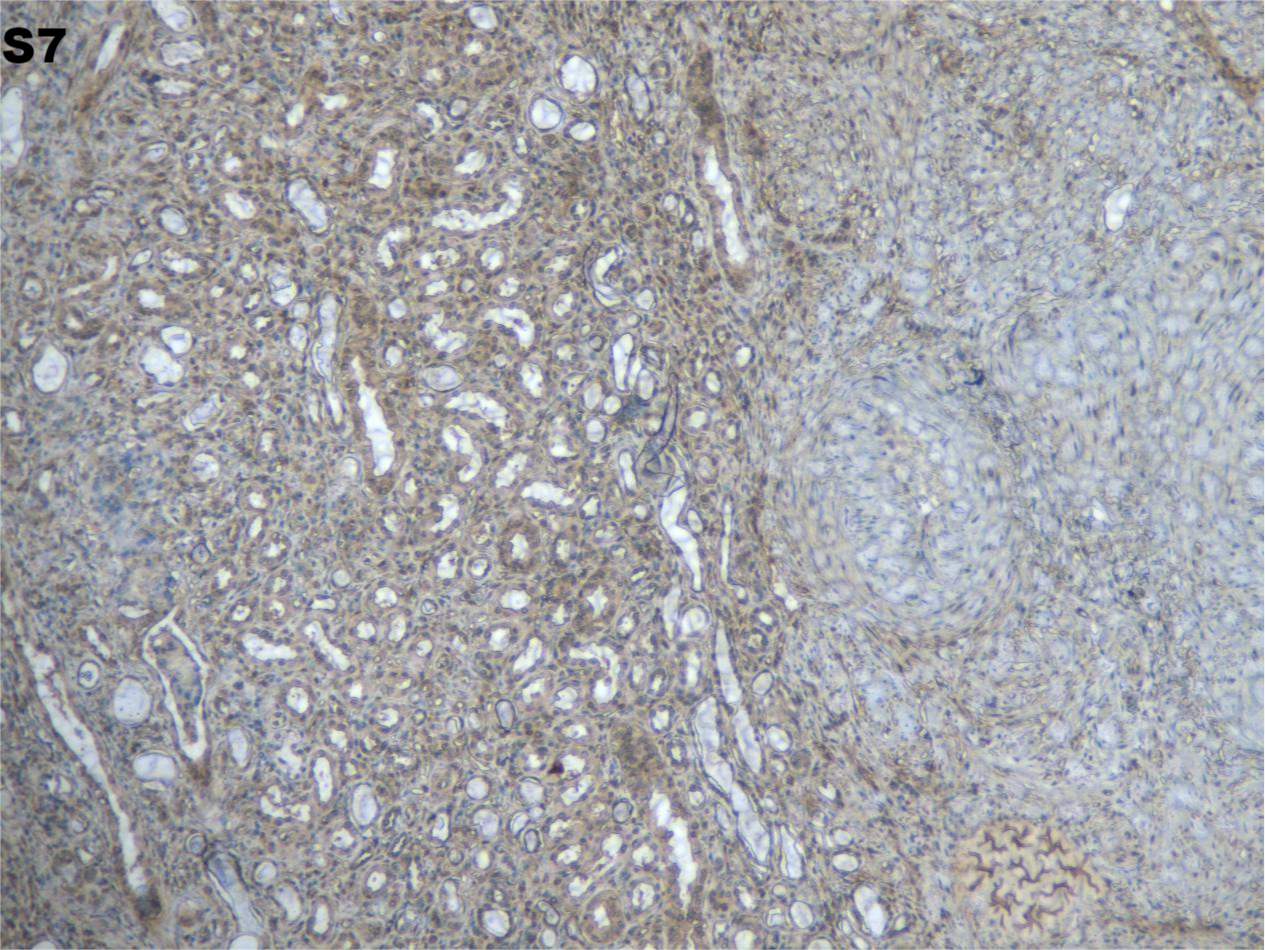


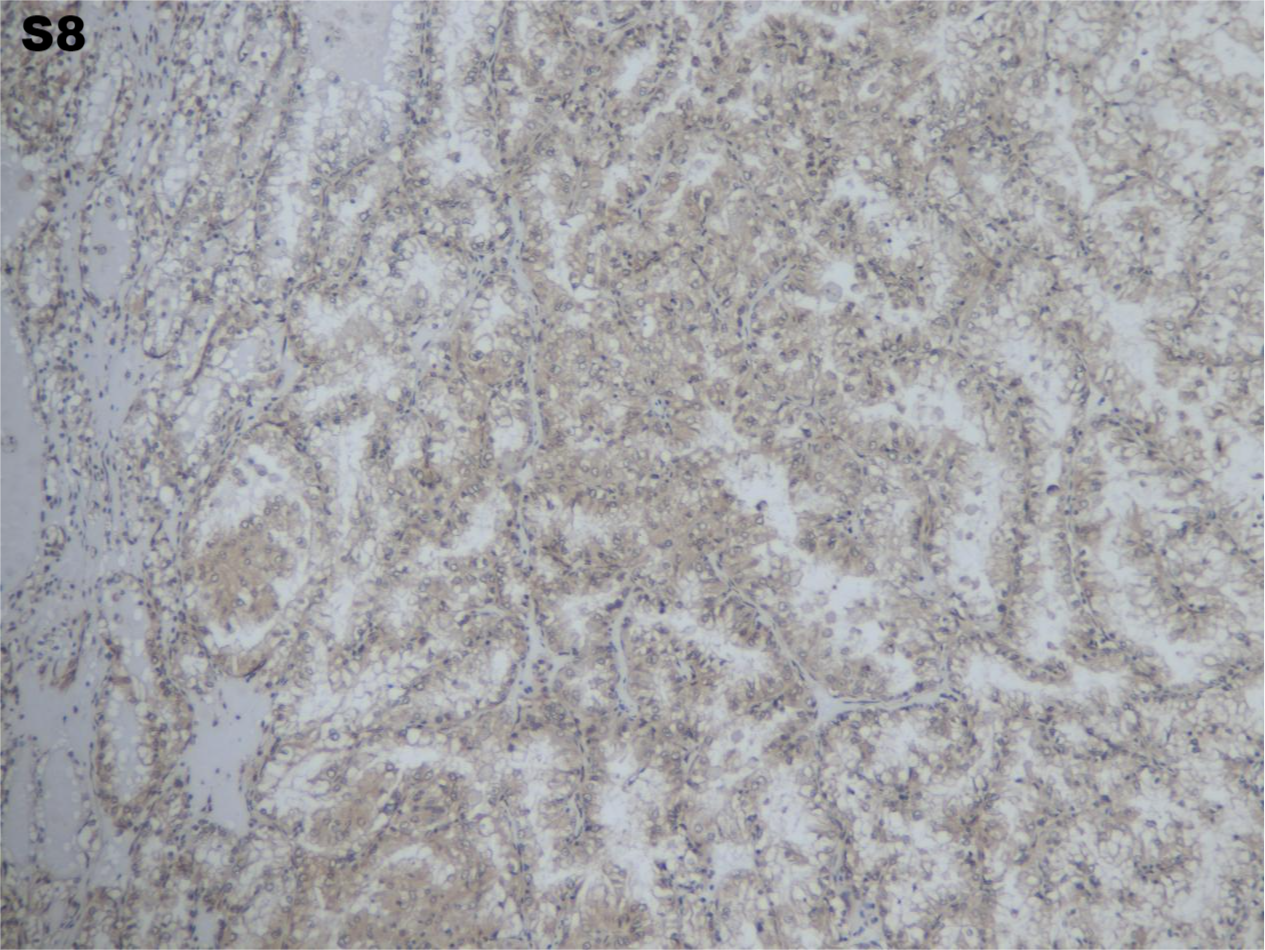


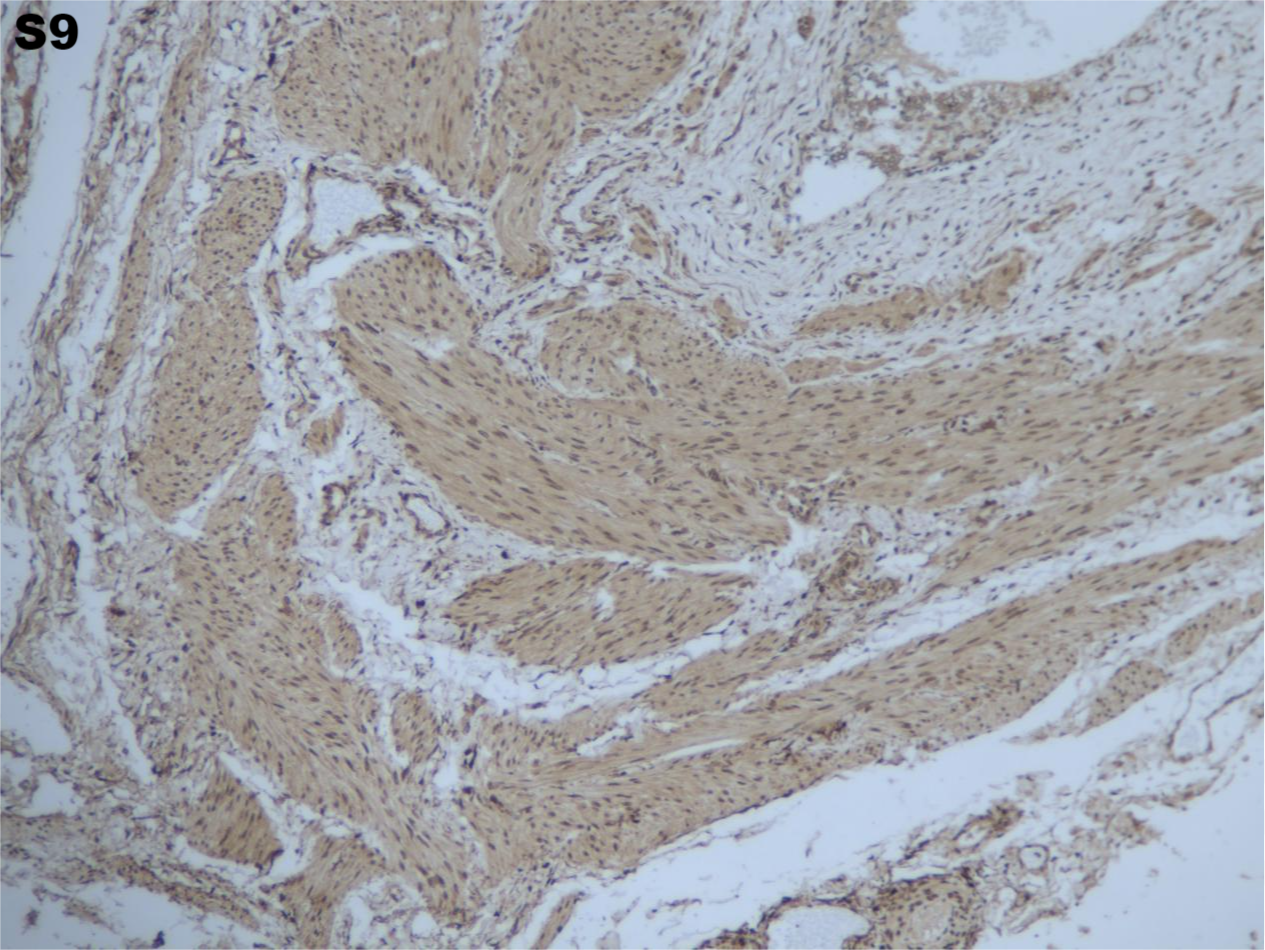


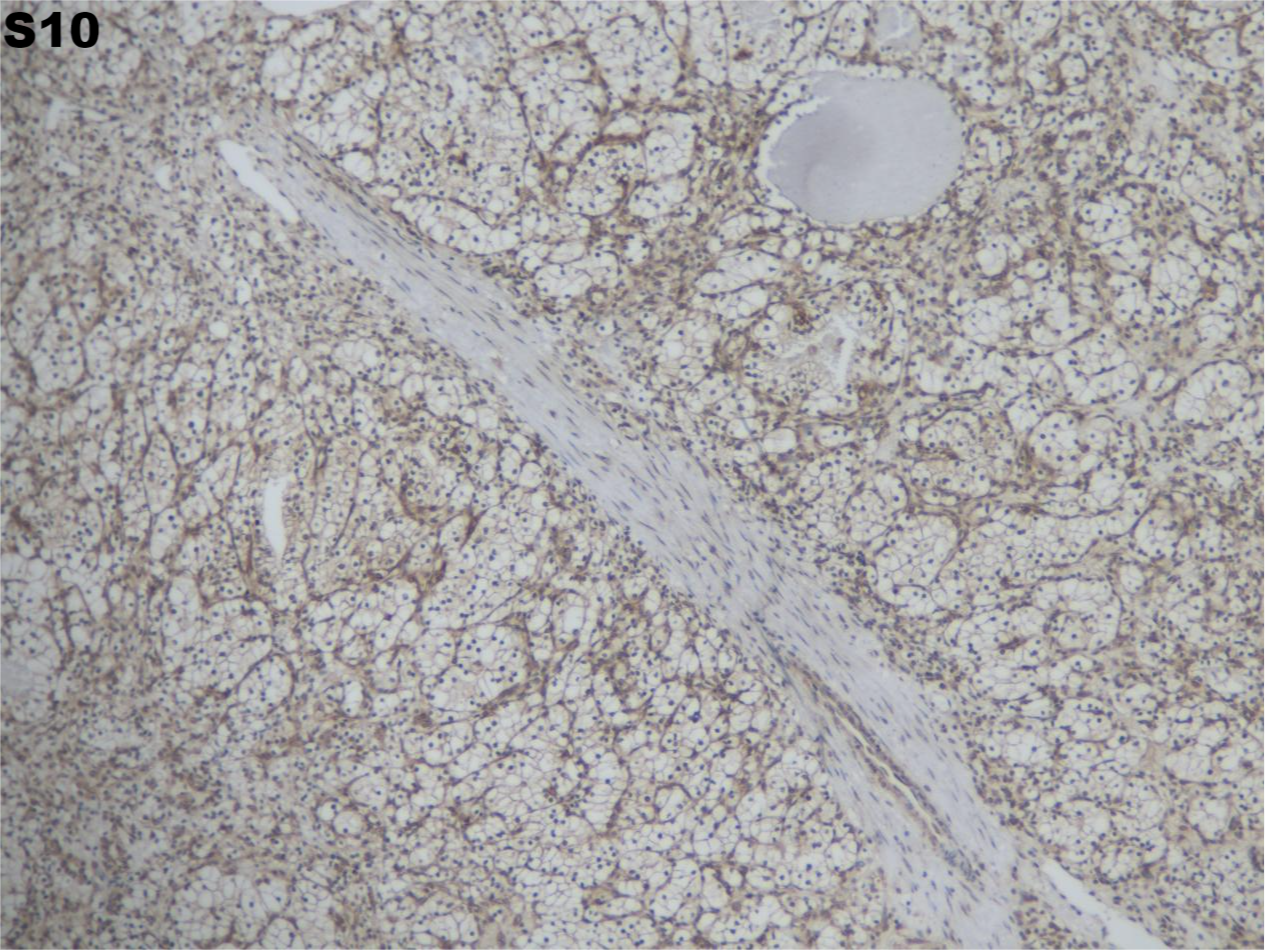

Supplement: Supplementary file 1 — Additional file 1. Data and analysis of this study. [file 12894_2022_1087_MOESM1_ESM.zip › Supplementary/Image of IHC(T)(1).docx]

Figure Legends

Image of IHC(Tumor)

Figure S11-17 The expression of AKT1 in tumor tissues.


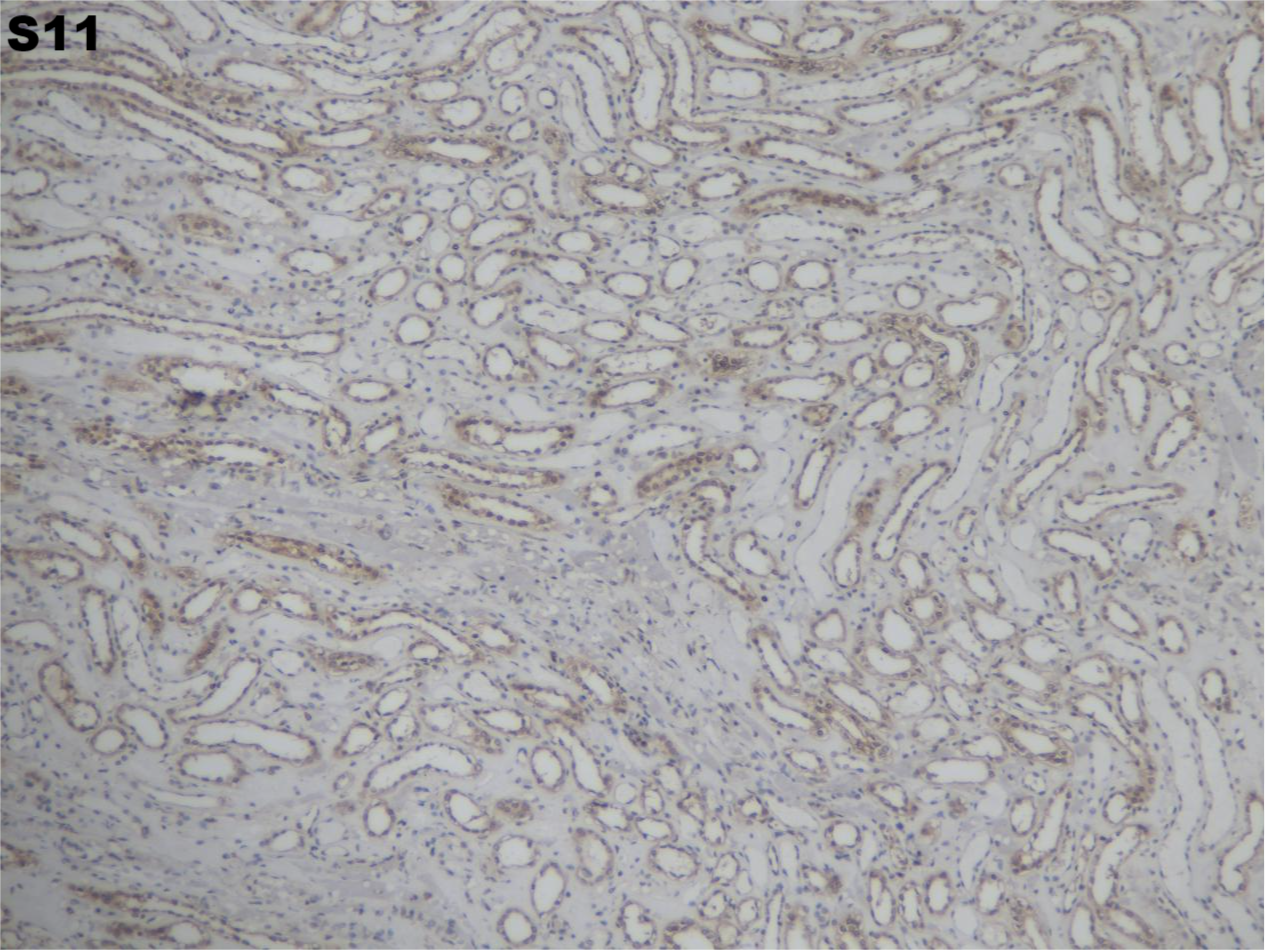


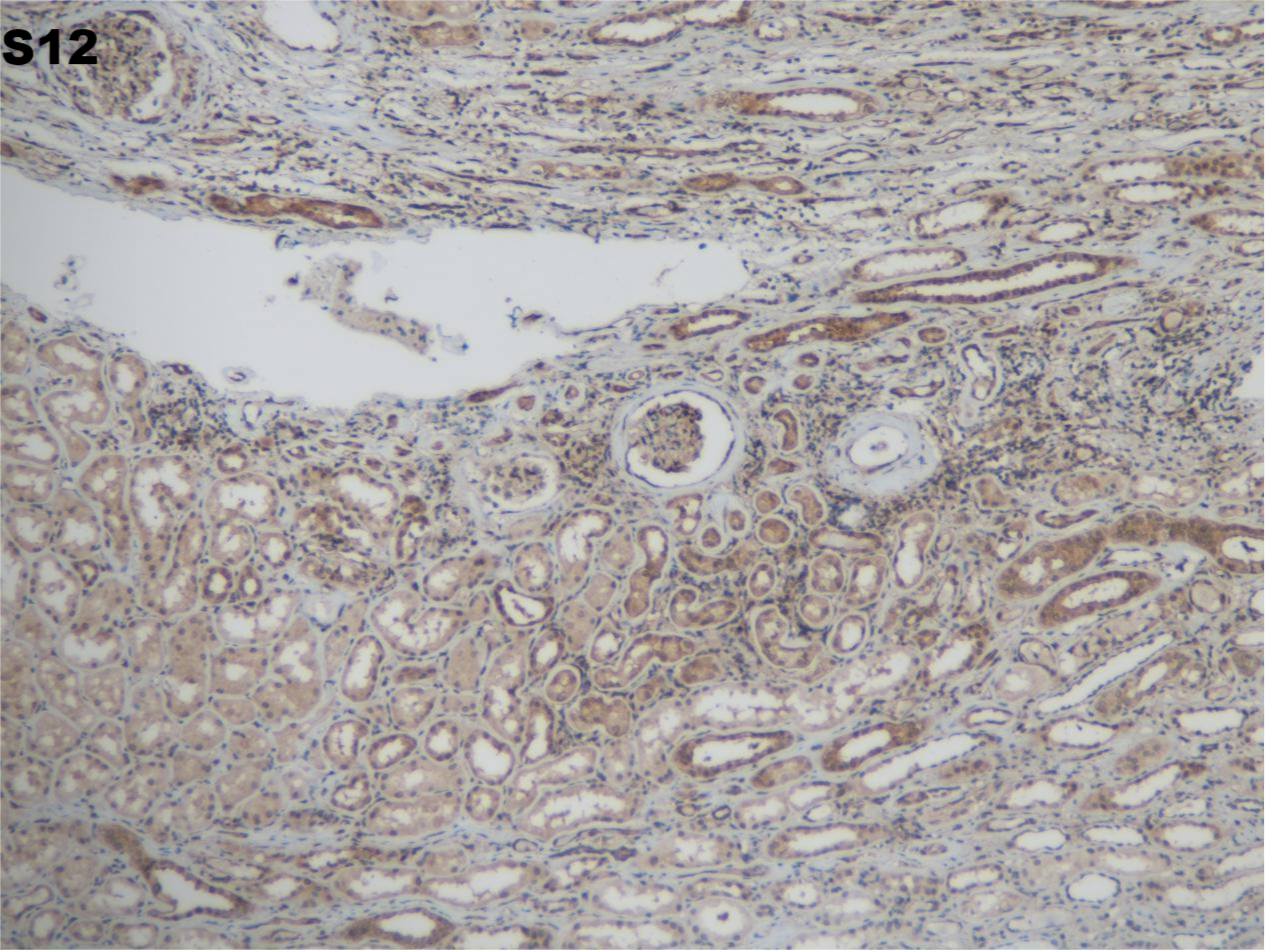


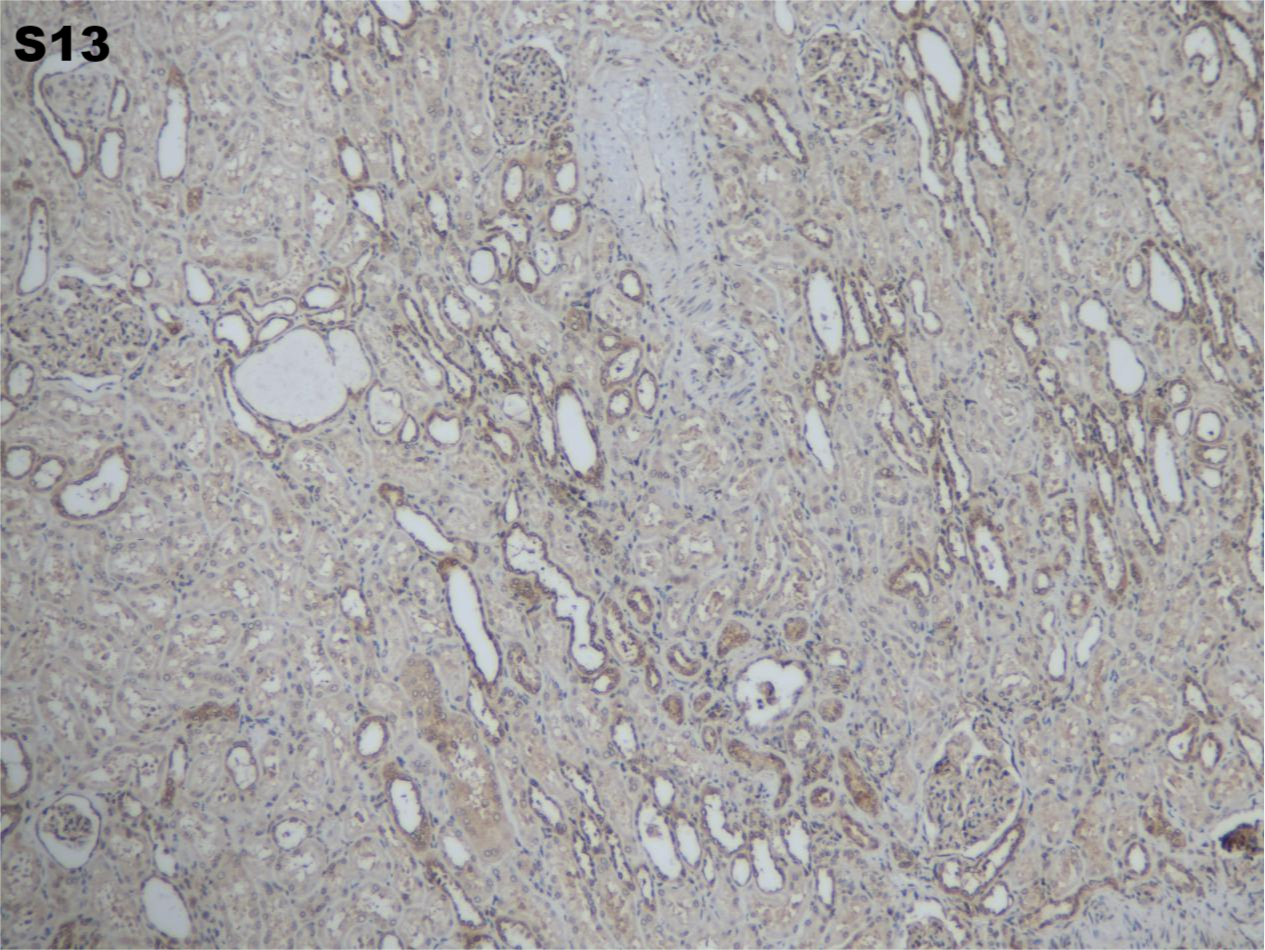


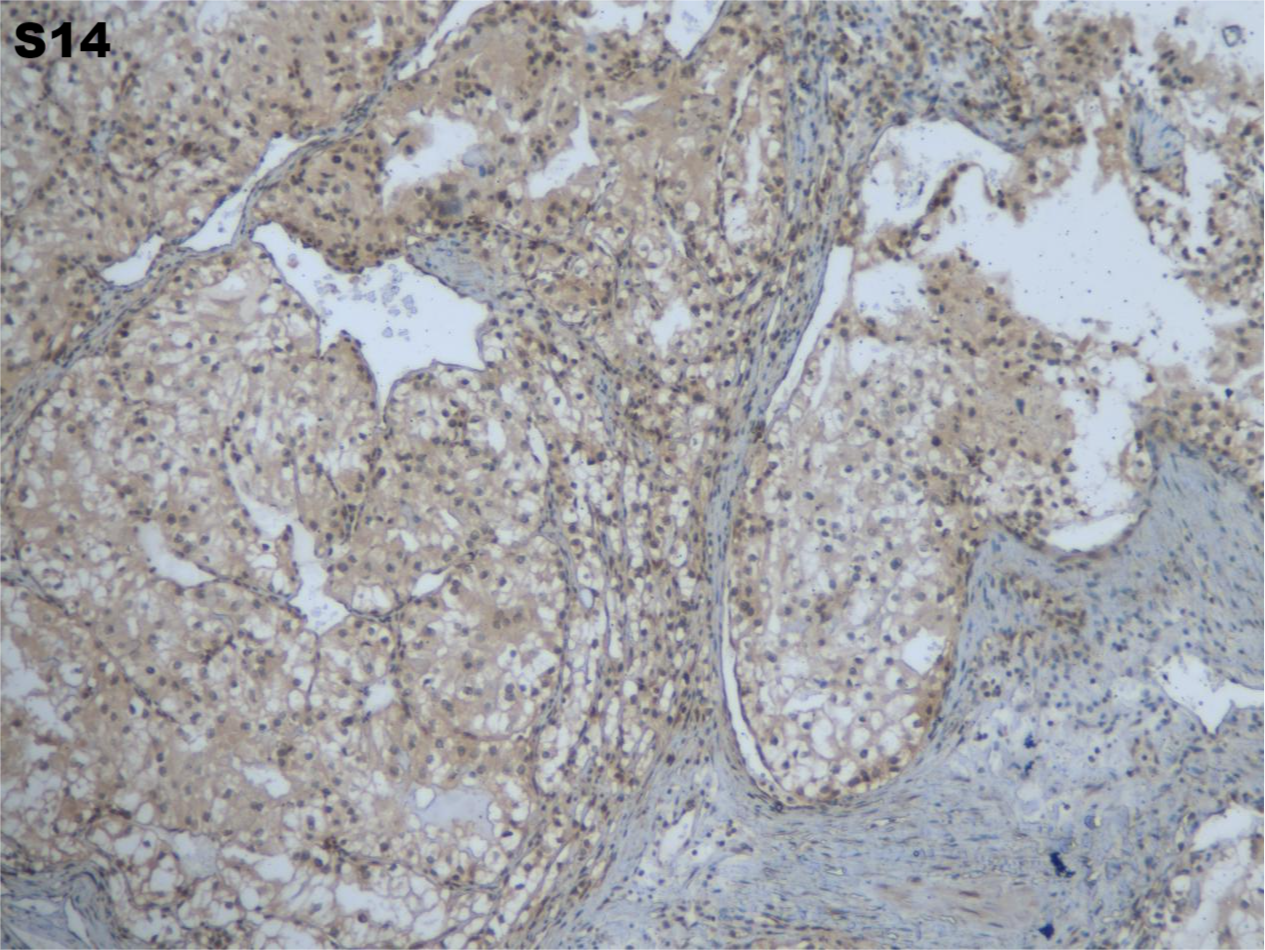


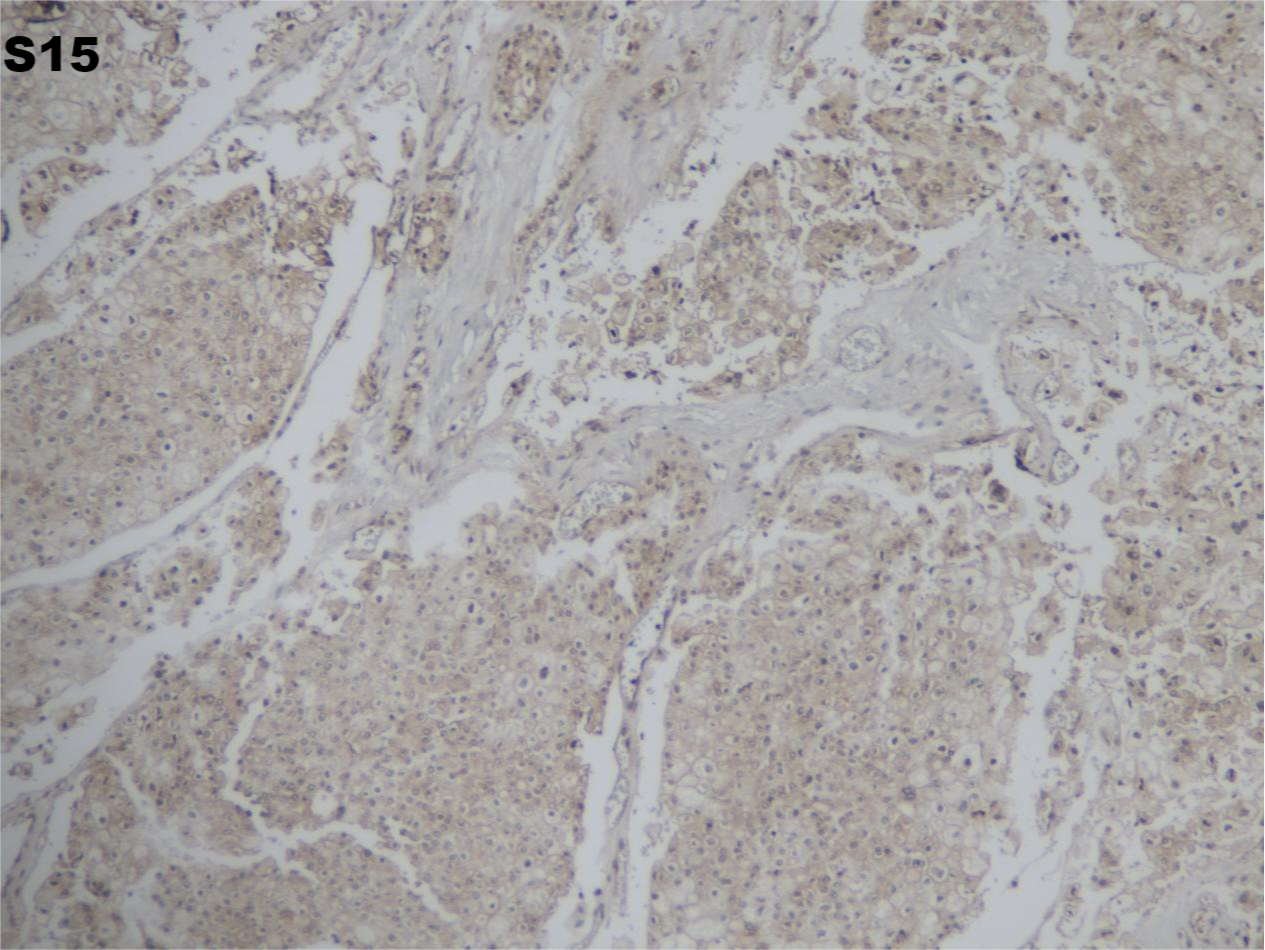


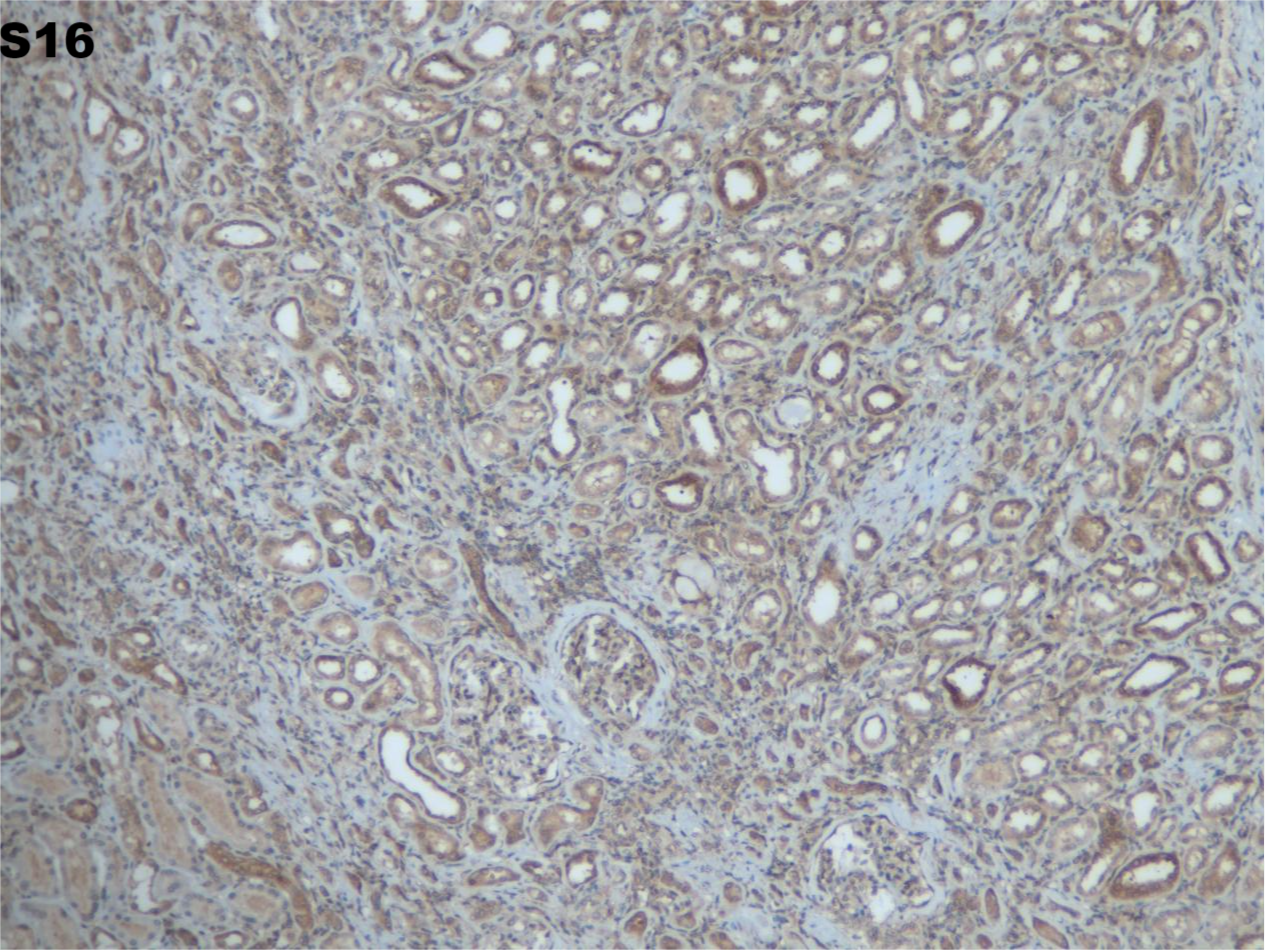


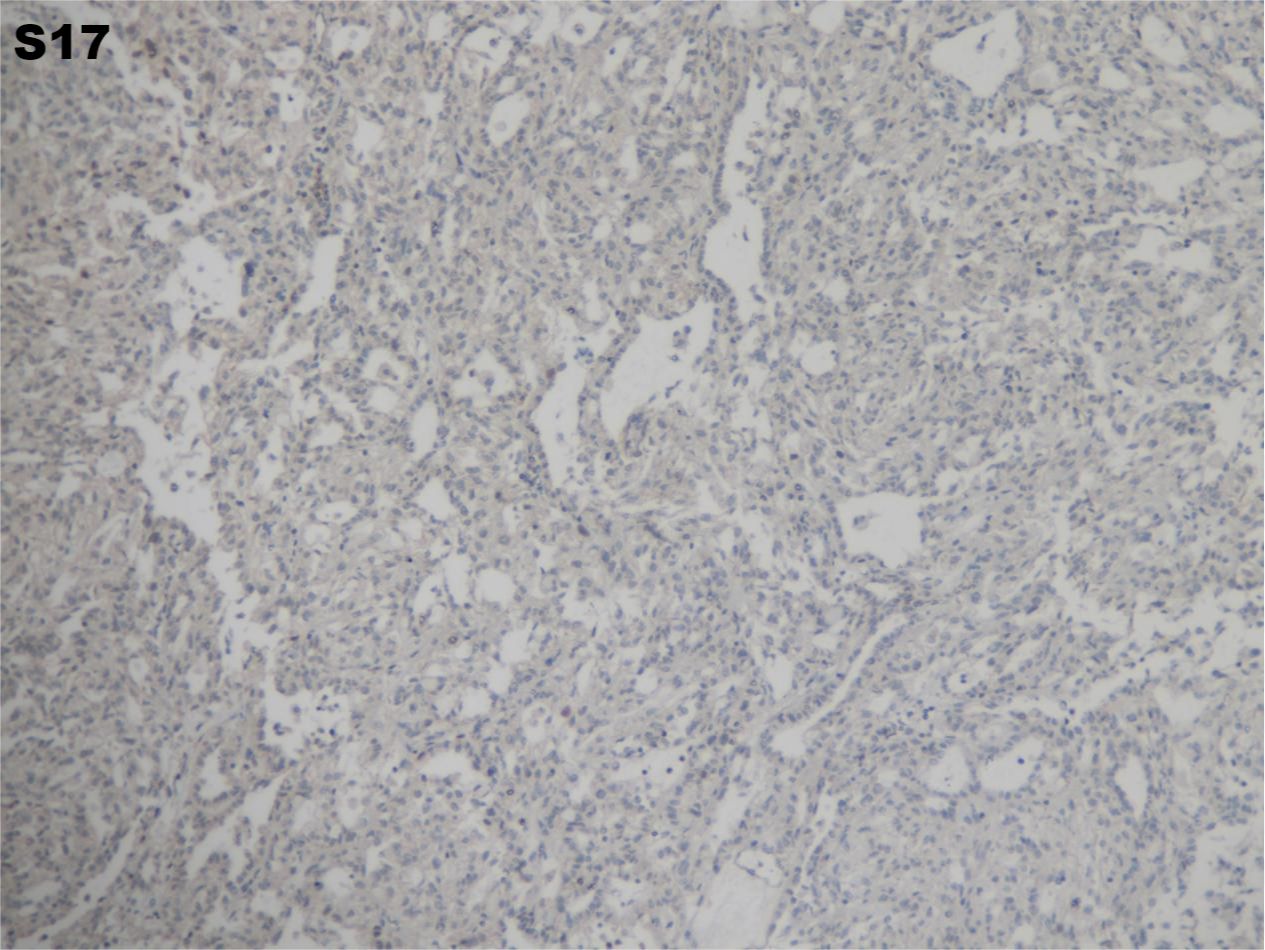

Supplement: Supplementary file 1 — Additional file 1. Data and analysis of this study. [file 12894_2022_1087_MOESM1_ESM.zip › Supplementary/Image of IHC(T)(2).docx]
