# Supplementary material for: Effect of AKT silence on malignant biological behavior of renal cell carcinoma cells
Source: BMC Urol. 2022 Aug 22;22:129. doi: 10.1186/s12894-022-01087-4 (PMC9396790; doi:10.1186/s12894-022-01087-4)
Supplement: Supplementary file 1 — Additional file 1. Data and analysis of this study. [file 12894_2022_1087_MOESM1_ESM.zip › Supplementary/cell image of Scratch test(1).doc]

Figure LegendS

Scratch test to determine cell migration.

Figure S1 cell image of control-1-0h. FigureS2 cell image of control-1-48h. Figure S3 cell image of control-2-0h. Figure S4 cell image of control-2-48h. Figure S5 cell image of control-3-0h. Figure S6 cell image of control-3-48h. Figure S7 cell image of control-4-0h. Figure S8 cell image of control-4-48h. Figure S9 cell image of control-5-0h. Figure S10 cell image of control-5-48h.
