# Supplementary material for: Effect of AKT silence on malignant biological behavior of renal cell carcinoma cells
Source: BMC Urol. 2022 Aug 22;22:129. doi: 10.1186/s12894-022-01087-4 (PMC9396790; doi:10.1186/s12894-022-01087-4)
Supplement: Supplementary file 1 — Additional file 1. Data and analysis of this study. [file 12894_2022_1087_MOESM1_ESM.zip › Supplementary/cell image of Scratch test(2).doc]

Figure LegendS

Scratch test to determine cell migration.

Figure S11 cell image of NC-1-0h. Figure S12 cell image of NC-1-48h. Figure S13 cell image of NC-2-0h. Figure S14 cell image of NC-2-48h. Figure S15 cell image of NC-3-0h. Figure S16 cell image of NC-3-48h. Figure S17 cell image of NC-4-0h. Figure S18 cell image of NC-4-48h. Figure S19 cell image of NC-5-0h. Figure S20 cell image of NC-5-48h.
