# Supplementary material for: Effect of AKT silence on malignant biological behavior of renal cell carcinoma cells
Source: BMC Urol. 2022 Aug 22;22:129. doi: 10.1186/s12894-022-01087-4 (PMC9396790; doi:10.1186/s12894-022-01087-4)
Supplement: Supplementary file 1 — Additional file 1. Data and analysis of this study. [file 12894_2022_1087_MOESM1_ESM.zip › Supplementary/cell image of Scratch test(3).doc]

Figure LegendS

Scratch test to determine cell migration.

Figure S21 cell image of siRNA-2-1-0h. Figure S22 cell image of siRNA-2-1-48h. Figure S23 cell image of siRNA-2-2-0h. Figure S24 cell image of siRNA-2-2-48h. Figure S25 cell image of siRNA-2-3-0h. Figure S26 cell image of siRNA-2-3-48h. Figure S27 cell image of siRNA-2-4-0h. Figure S28 cell image of siRNA-2-4-48h. Figure S29 cell image of siRNA-2-5-0h. Figure S30 cell image of siRNA-2-5-48h.
