# Supplementary material for: Effect of AKT silence on malignant biological behavior of renal cell carcinoma cells
Source: BMC Urol. 2022 Aug 22;22:129. doi: 10.1186/s12894-022-01087-4 (PMC9396790; doi:10.1186/s12894-022-01087-4)
Supplement: Supplementary file 1 — Additional file 1. Data and analysis of this study. [file 12894_2022_1087_MOESM1_ESM.zip › Supplementary/cell image of Transwell(3).doc]

Figure Legends

Transwell assay to determine cell invasion.

Figure S9-10 cell image of siRNA 2. Figure S11-12 cell image of siRNA 3.
